# Engineering *in vitro* human neural tissue analogs by 3D bioprinting and electrostimulation

**DOI:** 10.1063/5.0032196

**Published:** 2021-04-02

**Authors:** Danielle Warren, Eva Tomaskovic-Crook, Gordon G. Wallace, Jeremy M. Crook

**Affiliations:** 1 ARC Centre of Excellence for Electromaterials Science, Intelligent Polymer Research Institute, AIIM Facility, University of Wollongong, Fairy Meadow, NSW 2519 Australia; 2 Illawarra Health and Medical Research Institute, University of Wollongong, Wollongong, NSW 2522, Australia; 3 Department of Surgery, St Vincent's Hospital, University of Melbourne, Fitzroy, VIC 3065, Australia

## Abstract

There is a fundamental need for clinically relevant, reproducible, and standardized *in vitro* human neural tissue models, not least of all to study heterogenic and complex human-specific neurological (such as neuropsychiatric) disorders. Construction of three-dimensional (3D) bioprinted neural tissues from native human-derived stem cells (e.g., neural stem cells) and human pluripotent stem cells (e.g., induced pluripotent) in particular is appreciably impacting research and conceivably clinical translation. Given the ability to artificially and favorably regulate a cell's survival and behavior by manipulating its biophysical environment, careful consideration of the printing technique, supporting biomaterial and specific exogenously delivered stimuli, is both required and advantageous. By doing so, there exists an opportunity, more than ever before, to engineer advanced and precise tissue analogs that closely recapitulate the morphological and functional elements of natural tissues (healthy or diseased). Importantly, the application of electrical stimulation as a method of enhancing printed tissue development *in vitro*, including neuritogenesis, synaptogenesis, and cellular maturation, has the added advantage of modeling both traditional and new stimulation platforms, toward improved understanding of efficacy and innovative electroceutical development and application.

## I. INTRODUCTION

Biomedical research is benefiting from innovative *in vitro* engineered live-human neural tissue modeling. The state-of-the-art tissue building is ever the more reproducible, accurate, accessible, and relevant for understanding healthy and pathological tissue development and function and prospective therapeutic potential.

Notwithstanding the value of conventional human *ex vivo* tissue studies and *in vivo* animal models, the extrapolation of findings to live-human *in vivo* processes is relatively limited.^1,2^ For example, human postmortem brain tissue only provides a snapshot of *in vivo* tissue function and cellular processes, while animal modeling of disease is often centered on a particular phenotype or partial underlying pathology, thereby failing to capture the whole spectrum of important processes or account for possible comorbidities. These limitations explicate some of the findings of comparative studies between homologous human and animal (e.g., murine) cell types that have observed extensive differences, ranging from alterations of the intrinsic membrane and electrical properties over altered laminar distribution to distinct gene expression and morphological variations.^3,4^ In addition to the significant ethical considerations, there is clearly a need for accessible and valid (biologically relevant) human neural tissue models with the ability to track normal and aberrant cellular and molecular interactions, as well as whole system interconnectivity.^2,5^ The recapitulation of cellular disease models is particularly challenging for heterogeneous diseases such as neuropsychiatric disorders (e.g., schizophrenia, autism, or bipolar disorder), with complex and variable phenotypes, where the underlying pathology and causation are not well understood.^5,6^

With these difficulties in mind, bioengineered neural tissue modeling is an appealing and increasingly viable alternative to traditional approaches. The aim is to emulate native (such as brain) tissue form and function, inclusive of intercellular interactions and networking, molecular signaling, and the natural extracellular microenvironment, for consistent and translatable experimental models. The composition and distribution of neurons and supporting cells and the extracellular matrix (ECM) are key features, affecting cell survival, source cell differentiation, migration, and whole tissue function.^7,8^

Until recently, two-dimensional (2D) cell culture models have been the predominant method for *in vitro* live-cell observation and manipulation of molecular processes and interactions, despite challenges and recognized limitations of adequately recapitulating *in vivo* cell tissue and disease phenotypes.^8–10^ Two-dimensional cellular monolayers provide a relatively simple and cost-effective approach to cell culture and research, although the direct contact of cells to the planar surface of a glass slide or culture plate entails possible exposure to anomalous chemistries and mechanical properties that alter the cell function and behavior, which can be retained even upon changing the substrate (“mechanical memory”).^8,11^ Cellular factors influencing migration and cellular extensions such as neurites of neurons are limited as they become homogeneously dispersed throughout the medium.^9^ Further alterations of intercellular connections and interactions, as well as their tissue specific architecture, can be observed, such as abnormal polarization and flattened morphology, which do not replicate the three-dimensional (3D) signaling and networking *in vivo*.^11–15^ Consequently, 2D cell culture has failed to effectively replicate the whole extent of disease pathology, again, for neuropsychiatric and other brain disorder modeling, as well as related drug treatment effects.^8,10–12,16^ In contrast, recent advances in the development of 3D neural cell models represent a significant improvement, more closely mimicking a cell's natural extracellular microenvironment, spatial distribution, and function or dysfunction.^17^ Furthermore, encapsulated neural cells in a 3D scaffold display accelerated differentiation, maturity, and synapse formation.^18–20^

As is the case *in vivo*, vital engineered 3D ECM enables accumulation and relevant distribution of nutrients and cellular metabolic discharges, with the structural support to allow for early complex 3D cellular interactions throughout the matrix. Not surprisingly, slight material variations of the biochemical composition of the 3D constructed ECM impact cell behavior and can be manipulated to guide aspects such as cell survival, differentiation, and maturation.^7,11^ Correspondingly, different 3D cellular models have been developed, ranging from the formation of spheroids from simple same cell clusters embedded within a matrix to similarly forming organoids from cell aggregations containing multiple differentiation states or cell types (e.g., mesenchymal and epithelial), as well as encapsulating stem cells in biomaterials for 3D casting or printing, followed by lineage specific induction.^20–23^ Notably, although spheroids or organoids resemble *in vivo* tissue more closely by recapitulating development and self-organization of cells, they are difficult to standardize for reproducible modeling, impacting reliability for definitively identifying disease pathology.^9^ Nonetheless, much effort is being made to improve methods of production through standardization and application of simpler and better-defined biomaterial-based systems toward simpler cell culture and differentiation.^24,25^

Progressive biofabrication of 3D cell models through 3D bioprinting aims to provide a more controlled cell and tissue construct, ideally created with biomaterials containing bioactive molecules, for precise spatial arrangement of cells within a scaffold.^26–28^ Further methodological placement of microcapsules within the cell-laden biomaterial can be used to refine the microenvironment through controlled release of biochemical factors, facilitating biochemical gradients that mimic developmental or disease-dependent environmental cues.^29^ The bioprinting technique and materials encapsulating the cells must be carefully considered and developed around the native environment of the relevant cellular tissue that is to be modeled. With the example of printing cells for neural tissues to model neuropsychiatric diseases in mind, recent genome-wide meta-analysis compiled from genome-wide association studies (GWAS) have highlighted the prevalence of shared risk genes amongst multiple disorders. The overlap of genes responsible for a particular clinical phenotype and shared symptomatic presentation^30^ emphasizes an underlying complexity, with 413 genic associations having been identified in schizophrenia (SZ), which are located across 13 brain regions,^31^ and 102 independent variants in 269 genes associated with major depressive disorder (MDD).^32^ Consequently, the cell source and 3D configuration of the cellular material employed for patient-specific modeling are extremely important, even before external risk-factors can be taken into account. The discovery of reprograming easily accessible human dermal fibroblasts into induced pluripotent stem cells (iPSCs) by Takahashi *et al.* was an important step toward bridging the gap, enabling patient-specific cell modeling of both mono- and heterogeneous diseases *in vitro* without prior knowledge of cellular pathology.^33^ The genic relevance, self-renewing, and pluripotent properties of iPSCs allow for expansion by culturing to large cell numbers and ensuing directed fate determination into disease relevant (neural) cell types for functional and morphological analyses to elucidate observed clinical phenotypes. The cells, therefore, are excellent candidates for bioengineering through 3D printing disease relevant human neural tissues.^5,26^ Notwithstanding the clinical relevance and compatibility with printing of human iPSCs for tissue modeling, individual variability concerning epigenetic traits such as DNA methylation should be considered, in addition to potentially related varied differentiation potential between iPSC lines.^5,34–36^

Although there are relatively few accounts of 3D bioprinting human iPSCs for engineering neural tissues, and despite the above-mentioned caveats, taken together with other examples of stem cell printing for neural tissue analogs, there is increasing evidence of improved recapitulation of known and novel *in vivo* disease characteristics whether engineered into cells and tissues or inherently expressed.^37^ There is, however, another potentially important caveat to printing iPSCs for tissue modeling, being conventional differentiation protocols often result in immature (foetal-like) tissues.^38–40^ Although being able to capture early neurodevelopmental aspects of disease, variants may be important for modeling disease-associated cellular phenotypes and treatment responses and modeling mature pathological characteristics found in patients, particularly with regard to studying progressive and later-stage neurodegenerative aspects of a disease. As such, there is a vital need for new methods to enhance cellular and tissue maturation to overcome the limitations of traditional protocols.

Recent *in vitro* studies of the use of electrostimulation for tissue engineering provide evidence for its novel application to promote and enhance stem cell proliferation and maturation of derivative neural cells and 3D tissues.^41–44^ From our own research relating to electrical stimulation (ES) for human neural stem cell (NSC) and iPSC differentiation, we have shown stem cell fate determination and guided differentiation toward neuronal cells without the use of exogenous chemical inducers.^45^ Although the cellular response to ES can vary due to the extracellular environment's limitation on ion flow, mode of charge delivery, cell-specific effects and other factors,^46–49^ the incorporation of ES into neural tissue building with 3D printing is promising for next generation modeling. It may also extend to clinical application in the form of electroceuticals for disorders such as SZ, MDD, and Parkinson's disease, in conjunction with printed tissues for tissue replacement therapy.^50–55^ Despite its long-established use as a clinical tool, including pacemakers for the heart, cochlear implants for the ears. and deep-brain stimulation for Parkinson's disease, the exploitation of ES *in vitro* has only recently begun. Also, the underlying bio-therapeutic mechanisms elicited by ES remain largely unknown.^56,57^ Integrated modeling through printing and stimulating tissue models will likely benefit both research and translational tissue application. For example, decreased neurite outgrowth and neuronal interconnectivity have been implicated in SZ pathology through both 2D and 3D cellular modeling, both of which are augmented by applied ES, supported by enhanced *in vitro* synaptogenesis.^42^

**FIG. 1.**
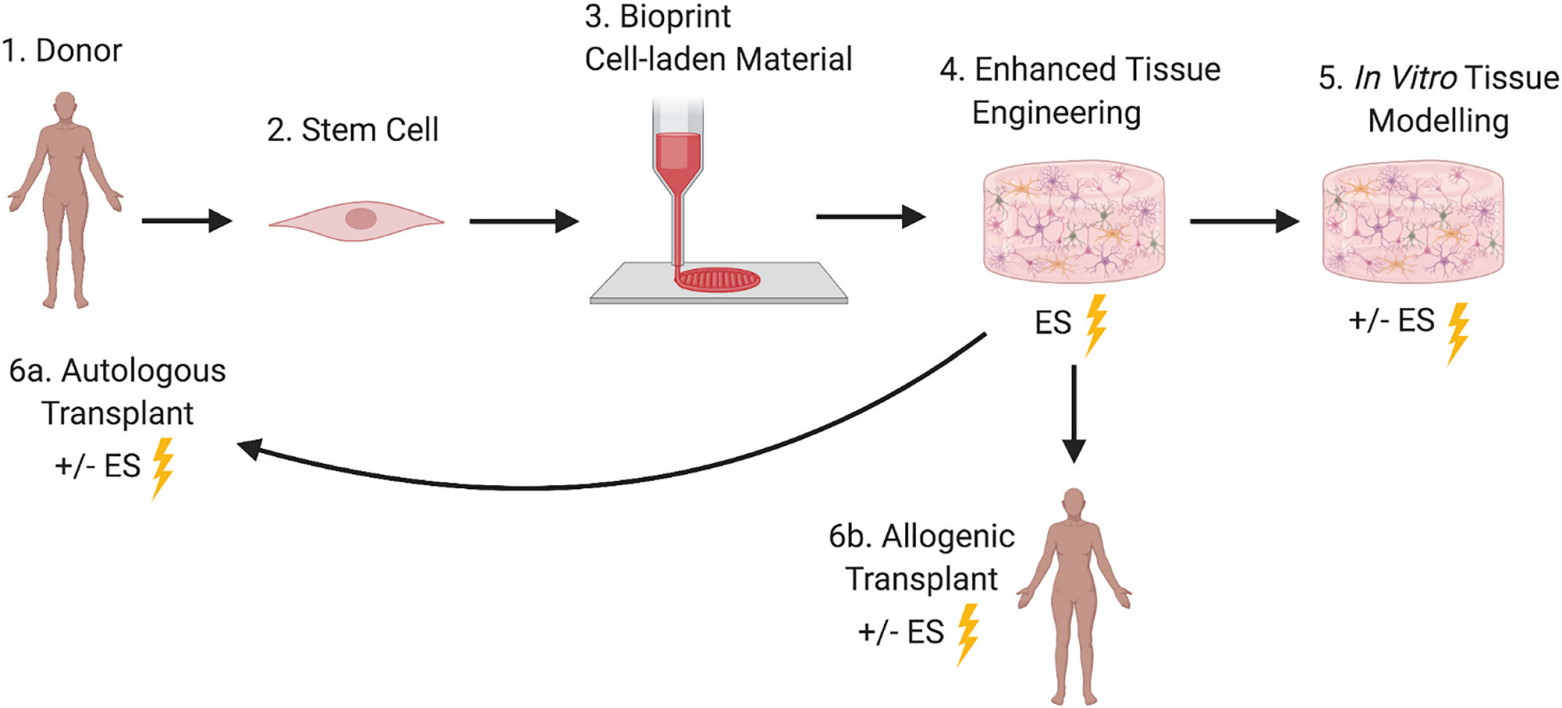
Schematic of three dimensional (3D) bioprinting and electrical stimulation (ES) of human stem cells for enhanced neural tissue engineering, *in vitro* tissue modeling, and clinical translation for tissue replacement therapy.

With a focus on 3D bioprinting to engineer and model native human neural tissues *in vitro*, this perspective article presents an overview of front-line techniques and experimental parameters for printing cells to form derivative tissue analogs. Progress toward standardized and reproducible models will be discussed, with emphasis on bioprinting iPSCs and NSCs of human origin and compatible biomaterials. Additionally, the promise of ES as a means to enhance printed tissue formation and maturation is also considered, with a view to promulgating the interfacing of 3D printed material-matrices and cells with modern bioelectronics for a most optimal and clinical compatible approach (Fig. 1).

## II. BIOPRINTING HUMAN STEM CELLS FOR NEURAL TISSUES

Bioprinting 3D *in vitro* cell cultures aims to replicate native tissue form and function, inclusive of the cellular microenvironment. The process begins by designing a printable 3D construct using computer aided design (CAD) software. A bioink comprising natural, synthetic, or semisynthetic biomaterial and cells is, then, printed in accordance with the design, enabling controlled temporospatial distribution of the ink, including encapsulated cells.^26,58^ The process is precise and controlled to enable more replicable and standardized 3D tissue building, including disease-relevant tissues for effective modeling, previously unattainable using traditional methods. Bioprinting neural tissues using human stem cells such as native NSCs or iPSCs requires several key considerations including the optimal printing technique, biocompatible material(s) (as the basis of a bioink) with appropriate rheological properties, and bio- and mechano-stability of printed constructs for ensuing stem cell culture, tissue development, and possible characterization.^27,59^ Importantly, the mechanical and structural properties of the printed scaffold determine the stability, as well as the shape fidelity of the construct, and guide cell proliferation, migration, and interconnectivity. A macroporous scaffold is necessary for cell maintenance, as it enables homogenous access to nutrients supplied by the culture medium.^60^ In addition, similar to shear, tension, and compression forces applied by native ECM, mechanical forces imparted by printed biomaterials act both intra- and extracellularly through various structural components, including the cellular membrane with cell surface receptors acting as mechanochemical transducers. Biophysical cues are, therefore, translated into biochemical signals through force-activated mechanisms involving the cytoskeleton, surface adhesion receptors (e.g., integrins), and nucleus-mediated transcription. Mechanosensitive mechanisms also include focal-adhesion signaling, actin-myosin contraction regulation, stretch activated ion channels, and force-sensitive activation of transcription factors.^61–64^ Integrins respond to topographical cues of the natural ECM (e.g., fibronectin and proteoglycans), facilitating focal adhesion (FA) activation, in addition to reorganization of the cytoskeleton (e.g., actin filaments).^64^ This results in phosphorylation of the mechanosensitive protein focal adhesion kinase (FAK) of the focal adhesion complex, which in turn activates the mitogen-activated protein kinase kinase / extracellular signal-regulated kinase (MEK/ERK) signaling pathway. MEK/ERK signaling has been associated with induction of transcription factors influencing neural differentiation, adhesion, and neurite outgrowth.^65,66^ Within this FA complex, another mechanosensitive protein, vinculin, reacts to mechanical strain and affects the cryptic kinase binding site for mitogen-activated protein kinase (MAPK). MAPK mediated signal transduction also underlies neural differentiation of mesenchymal stem cells (MSCs).^67^ Another mechano-mediated mechanism involves activation of Ras homolog gene family member A (RhoA), through membrane receptors (e.g., N-cadherin and integrins), and acts on downstream effectors, which modulates the actin cytoskeleton organization and promotes FA formation and stress fiber (actin-myosin) assembly. Its activation of Rho-associated protein kinase (ROCK) promotes neurogenesis, migration, neuronal differentiation, and focal adhesion of stem cells, again in part due to consecutive activation of the MEK/ERK pathway; however, inhibition of ROCK through exposure to compression forces can lead to neural apoptosis.^46,68–70^ Mechanically activated ion channels on the other hand are directly gated by mechanical forces that induce a conformational change and subsequent channel opening (activation), allowing an influx of ions (e.g., Ca^2+^).^71^ Ionically induced downstream signaling cascades regulate transcription factors associated with neural behavior, including differentiation and neurogenesis.^72^ Topographical and force-mediated cues are, therefore, not only an essential consideration for early stem cell viability but provide cellular cues that guide cellular fate, alignment, and morphogenesis.^46,61,63,64,73^

Cell–cell interactions also influence the cellular response to physical and chemical cues presented by scaffolds, including counteracting forces across cellular junctions that provide contact or adhesion between neighboring cells or between a cell and the bio-material. Given the roles of cell junctions, it follows that the cellular interconnectivity is important for reducing stress placed upon cells and regulating other extracellular effects on, for example, cell fate.^74^ In the case of pluripotent stem cells, this guided development echoes embryonic development involving differentiation of pluripotent stem cells into the three germ layers (endoderm, ectoderm, and mesoderm) and ongoing cellular patterning to form higher order structures,^75,76^ with mechanical forces and biochemical cues of the ECM persisting to shape neuron and glial cell development from the ectoderm.^77^

The following section considers bioprinting techniques and parameters for both high initial post-printing stem cell viability within bioink sols and ensuing optimal mechanical and biochemical components of gelated constructs for stem cell differentiation into relatively complex functional 3D neural tissue models. Importantly, despite the variety of printer systems and strategies, several examples have emerged, which push the boundaries of biofabrication to build larger, more sophisticated tissue structures displaying salient features of native neural tissues.

### A. Bioprinting techniques

Bioprinting techniques and subvariants can be broadly categorized into (1) extrusion printing, (2) laser printing, and (3) inkjet printing.^26^ Bioprinting enables construction of a 3D CAD designed scaffold from a cell-laden bioink and is dependent on the material composition and related physicochemical properties (e.g., viscosity and cross-linking ability/requirements). The technique and printer components, such as the printing nozzle of inkjet and extrusion printers and laser intensity of laser printers, in conjunction with the bioink regulate shear stress and the cytotoxic radiation effect applied to cells; this in turn governs post-printing cell viability and morphology, depending on the cell type and innate sensitivity.^26^ For stem cells, the mechanical force experienced during and after printing can initiate the previously discussed mechanosensitive mechanisms that impact cell behavior, affecting cell proliferation and specification. This is consistent with stem cells being regulated by both intrinsic and extrinsic forces during *in vivo* development.^73^ This underlines the importance of precise monitoring and control of optimized parameters during the construction of the cell-laden scaffold. Both cell viability and scaffold stability (mechanical and chemical) are priority considerations and are dependent on the characteristics of the chosen biomaterial, including viscosity, gelation kinetics, and modulus, with the latter influencing neuritogenesis and neural networking. The material composition also dictates the choice of printing method, such as temperature during printing, which will in turn influence the rate of ink transfer and achievement of continuous and even live-cell-laden ink deposition for a uniformly layered scaffold. Thus, different materials and different techniques of bioprinting present strengths and weaknesses for optimal neuronal tissue building and modeling, which are further discussed below.^78^

Methods for printing neural tissues derived from iPSCs principally entail two approaches. First, bioprinting pre-differentiated neural progenitor cells is most common, with fewer accounts of more difficult iPSC printing, with the latter extending to *in situ* formation of embryoid bodies (EB) prior to differentiation.^79,80^ A potential advantage of pre-differentiating cells prior to printing is greater control of differentiation outcome and more precise determination of the desired ratio of specific neural cell types in the printed construct, as well as their placement.^81^ However, the experimental outcomes of printing iPSCs for neural tissues support biofunction akin to native tissue, through the formation of 3D neural networks of both neuronal and glial cell types, displaying mature functional properties.^26,79,80^ Differentiation prior to printing would require subsequent separation of self-organized cellular connections, possibly counteracting the benefits of controlled replication of the *in vivo* heterogeneity and cell subtype ratio.

Most recently, extrusion printing in particular has been shown to be amenable for both pre- and post-printing differentiated iPSC-neuronal tissue models, although other techniques have also been successfully applied, including laser printing, stereolithography, and microfluidics-based printing, of which the 3D printed neural models constructed using human cells were compared (Table I).^20,79,82–87^ Other forms of bioprinting have been utilized to print pluripotent stem cells or a combination of different cell suspensions, for example, inkjet—piezoelectric printing, although these were not followed by differentiation to neuronal constructs.^26,88–90^

**TABLE I.** Methods and hydrogel components for 3D bioprinting human neural cellular systems. Relevant articles ordered by date, excluding research outcomes resulting with no consistent 3D scaffold and nonneuronal cell types. Assessments of cell viability range from 1 to 10 days post printing. NPC = neural progenitor cells, NSC = neural stem cells, iPSC = induced pluripotent stem cells, NA = neural aggregates, OPC = oligodendrocyte progenitor cells, GelMA = gelatin methacrylate, and GEL/FIB = gelatin mixed with fibrin.

Year	Ref.	Cell type	Printing technique	Materials	Cell viability %
2020	Sharma *et al.*^82^	NPC	Microfluidics-based	Fibrin, alginate, chitosan, calcium chloride, thrombin, genipin	∼90–95
2019	Fantini *et al.*^83^	NSC iPSC Neuroblastoma	Extrusion printing	Alginate, gelatin	∼100
2019	Abelseth *et al.*^85^	NA	Microfluidics-based	Fibrin, alginate, chitosan, calcium chloride, thrombin, genipin	∼85–95
2019	Salaris *et al.*^79^	Cortical neurons, glial cells	Extrusion printing with microfluidic printhead	Matrigel, alginate	∼70–80
2018	De la Vega *et al.*^86^	NPC	Microfluidics-based	Fibrin, alginate, chitosan, calcium chloride, thrombin, genipin	∼80–95
2018	Joung *et al.*^87^	NPC, OPC	Extrusion printing	GelMA, GEL/FIB, Matrigel	∼75
2018	Ho *et al.*^84^	Neural crest cells	Extrusion printing	Polyurethane (PU)	∼65
2017	Gu *et al.*^80^	iPSC	Extrusion printing	Alginate, carboxymethyl-chitosan, agarose	high
2016	Gu *et al.*^20^	NSC	Extrusion printing	Alginate, carboxymethyl-chitosan, agarose	∼75–90

#### 1. Extrusion printing

**FIG. 2.**
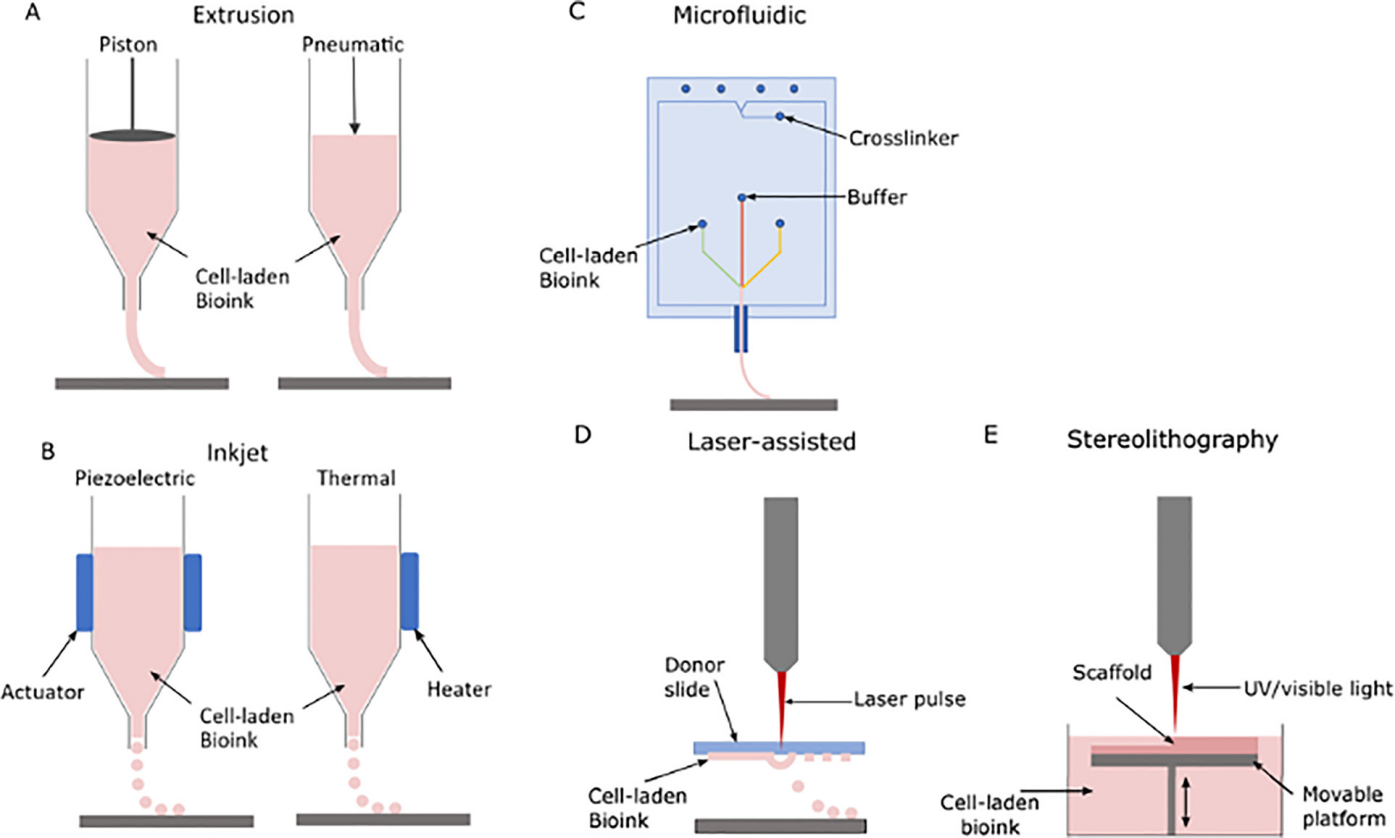
Schematic of selected bioprinting techniques and their components. (a) Extrusion printing, (b) inkjet printing, (c) microfluidic-based extrusion printing, (d) laser-assisted printing, and (e) stereolithography.

Extrusion of cell-laden bioink from the nozzle of a syringe by means of pneumatic or mechanical (piston or screw-directed) pressure is the underlying mechanism of extrusion printing (Fig. 2). Preprogramed deposition along the axes allows for controlled spatial arrangement of the bioink. The overall printing resolution of this technique is dictated by the nozzle diameter, ink viscosity, and speed of extrusion and remains relatively low (∼100–300 *μ*m) by dispensing larger volumes in comparison to other printing techniques.^78,91–93^ Scaffold production with comparatively higher bioink viscosities is also enabled by the extrusion pressure applied. Increased viscosity allows continuous flow and even ink distribution and can account for modeling with biomaterials containing higher cell densities.^9,94^ Adaptation for softer gels can easily be achieved by increasing the bioink's ejection speed, although this may compromise the scaffold stability and increase the extrusion-associated shear stress on cells.^91^ Further modification of this method to co-axial extrusion facilitates layer-by-layer printing of multiple materials within the same scaffold.^95^ The length of a syringe nozzle has also been found to affect cell viability, with shorter nozzles producing relatively less shear stress on cells and higher post-printing viability.^89^ Cell viability is affected by a number of interrelated printing parameters, such as pressure applied to expel a cell-laden ink from a nozzle, optimally set to also promote even distribution for scaffold formation. Low pressure (∼45–70 kPa) has been demonstrated to produce high cell viability of close to 100%, whereas higher pressure (>190 Pa and ensuing higher shear stress) reduces cell viability to <65% (Table I).^83,84^ Not surprisingly, cell proliferation correlates with increased cell viability following printing, resulting in a high cellular content of printed constructs. Notwithstanding the potential pitfalls of pressure on cell viability and morphology for extrusion printing, it remains one of the most common printing techniques, enabling the printing of a large variety of cells (Table I).^20,58,83,91,92^

##### a. Microfluidics-based printing for “lab on a chip” modeling

As a derivative of extrusion printing, microfluidics-based bioprinting for “lab-on-a-chip” modeling encompasses a platform containing individual materials and cell-laden microchannels that are merged before a combined bioink extrudes out to create a layered scaffold (Fig. 2). The separate channels allow controlled introduction of specific material components that can be secreted at different flow rates throughout the process.^9,86,96^ This pre-print material processing enables the designed scaffold to contain different gradients of channel components, introducing local variation of the bioink within the construct while maintaining even cellular distribution. Such fine-tuning allows for precise specification of the microenvironment, which can guide differentiation and influence cell behavior, better mimicking native tissue.^85,86^ The convergence of microchannels reduces shear stress on cells by encapsulating the cell-laden bioink with a cross-linking material before extrusion, supporting consistently high cell viability in printed scaffolds (80–95%).^82,85,86^ This ability to control fluid flow parameters through the channels allows for low pressure exposure of the bioink (20–50 mbar) and an adjusted higher pressure of buffer and cross-linking material (40–100 mbar), further decreasing shear stress on cells and reducing printing-dependent limitations on gel viscosity.^82,85,86^ Post-printing scaffold fidelity is dependent on material viscosity and the speed at which initiation of material cross-linking occurs. Notwithstanding, instantaneous release of, for example, an alginate bioink and its cross-linking agent calcium chloride (CaCl_2_) is possible during microfluidic printing, mitigating the effect of dispersion of low viscosity material prior to cross-linking and subsequently retaining scaffold fidelity. On the other hand, the addition of CaCl_2_ after extrusion printing of the scaffold through co-axial extrusion or manually increases the risk of bioink dispersion before cross-linking occurs. The Willerth Laboratory was the first to successfully print neural progenitor cells (NPCs) and neural aggregates (NA) derived from human iPSCs using a microfluidics-based RX1 Bioprinter (Aspect Biosystems).^82,85,86^ With a resolution of ∼100–200 *μ*m, this method depends upon the nozzle diameter for its resolution, similar to extrusion printing.^86^

#### 2. Inkjet (drop-on-demand) printing

Inkjet printing is an inexpensive and high through-put printing platform that requires low dynamic-viscosity (<10 mPa s) bioink for the creation of 3D scaffolds and relies on biomaterial surface tension.^78,91,97,98^ The low viscosity dictates the need for rapid cross-linking upon printing of biomaterials in order to maintain the scaffold structure.^94^ There are two different kinds of inkjet printing, namely, thermal and piezoelectric (Fig. 2). For thermal inkjet printing, an electrically heated element produces a localized vapor bubble within the bioink, which results in ejection of ink droplets from the nozzle through mechanical pressure. The droplets form a predesigned 3D scaffold in a layer-by-layer fashion. In place of the heated element, piezoelectric printing employs a piezoelectric actuator that generates acoustic waves. Similar to the vapor bubble of thermal inkjet printers, the acoustic pulse causes compression of the cell-laden bioink, forcing it through the nozzle as droplets that are again distributed on the printing platform to create a layered scaffold.^91,99,100^

Both types of inkjet printing are limited by the rheological properties of the printed material, requiring softer bioinks to fabricate biomimetic structures, potentially creating a challenge for consistent and stable layering of 3D scaffolds along the z-axis to form vertical structures and restricting the cell density within the bioink.^78,81,101^ As such, cross-linking polymers are an important component of the bioink in order to immediately solidify the construct on a layer-by-layer basis for a consistent build.^91,92^ The ability to dispense single small-volume (1–100 pL) droplets enables high-resolution printing, with the possibility of controlling cellular distribution on a single-cell level.^91,92^ However, the mechanical force, shear stress, and high temperatures (100–300 °C) are concerning for cell viability, formation of neural networks, and cellular function. Nonetheless, high cell viability (∼90%) is achievable.^9,92,99^ Overall, the major benefit of inkjet printing is the ability to precisely place small volumes of cell-laden bioink for higher print resolution (10–100 *μ*m) compared to most other printing techniques.^78,91,93^

#### 3. Laser-assisted printing

Laser-assisted printing is a nozzle-free printing method, which can be used to produce high resolution models (10–50 *μ*m).^81,91,92^ It requires a donor slide (ribbon) comprising a thin laser energy-absorbing layer above a thicker cell-laden bioink, which is placed underneath the laser. A parallel placed receiving substrate below the ribbon presents the platform for the bioprinted scaffold. Based on laser-induced forward transfer (LIFT), the pulse omitted by the laser pen causes evaporation of the upper ribbon layer to form a vapor bubble that exerts pressure on the bioink. As a result, localized micrometric droplets are transferred to the receiving substrate.^26,91,99^ This complex design mechanism allows layer-by-layer distribution of cell-laden bioinks along a predesigned 3D scaffold with single-cell specificity within pico-nanolitre printing volumes, providing high resolution and cellular organization.^78,91,92^ As the bioink does not have to be extruded through a nozzle, there is no mechanical stress imposed upon the encapsulated cells, allowing for a high cell viability of ∼95% in printed constructs.^26,58,78^ Although the laser-induced heat that the cells are exposed to can potentially cause cellular damage, it is optimized to be insignificant.^78,102^ The resolution of the printed scaffold is dependent on the droplet diameter, which can be manipulated through gel viscosity and the laser energy the ribbon is exposed to and can lie in the range of 1–300 mPa·s.^103^ Higher gel viscosity requires higher laser energy for the transfer of a larger diameter droplet onto the receiving substrate. This not only affects the printing resolution but also determines printing speed and is another important consideration for ensuring cell survival and even bioink distribution within the scaffold.^103^

Given the complex design and range of parameters, biomaterials of various viscosities are able to be printed, although the relatively slow printing speed (∼5 Hz) and high cost have limited the implementation and uptake of laser-assisted printing.^58,78,81,91,103^

##### a. Stereolithography

Stereolithography (SLA) is a laser-based printing technique for fast and precise production of high-resolution 3D bio-scaffolds.^26,104^ Photocuring with low-power ultraviolet (UV) or visible light enables patterned polymerization of photosensitive solutions.^92,100,104^ A movable platform submerged in bioink can be raised to the surface layer and exposed to a reflected UV laser, initiating polymerization and subsequent solidification of the targeted upper material layer (Fig. 2).^105^ A variety of materials can be printed using this technique, allowing for multifunctional scaffolds by incorporating biocompatible components with varying material properties and stiffness, provided that the bioink maintains its photocurable properties.^106^ Although post-printing cell viability is relatively low (up to 80%; Table I), compensatory cellular proliferation within the scaffold can continue after printing, as for other printing techniques.^26,107^ Cell death during the printing process can be caused by intense UV exposure and cross-linker associated cytotoxicity.^81,99^ However, cellular damage associated with shear stress is eliminated by being nozzle-free for scaffold construction, which also reduces nozzle-associated resolution limitations.

Scaffold resolution and printing accuracy in SLA rely largely on laser-dependent factors, such as laser power, scanning speed, laser spot size, and wavelength, and can be between 5 and 300 *μ*m.^92,105^ Taking these factors into account, SLA enables construction of scaffolds with homogeneous pores and has demonstrated even cellular distribution.^107,108^ Furthermore, the maintenance of mechanical strength has been achieved when combining multiple materials and cell types into a single 3D printed construct.^106,109^ Such control of material and resultant scaffold properties, including porosity, have enabled 3D printing of neuronal scaffolds using mouse cell lines.^107–109^ SLA in combination with electrospinning fibers has also enabled improved neurite formation in stiffer materials, demonstrating the technique's versatility and adaptable modeling potential.^109^

### B. Biomaterials

One of the main challenges that 3D bioprinting presents is the requirement of biocompatible materials that recapitulate the properties of native ECMs following printing. In the central nervous system (CNS), intracellularly biosynthesized ECM components are secreted by neuronal and glial cells and aggregate to form the ECM.^110,^^111^ The components surround and interact with the neural cell membrane, including integrin receptors, which influences signaling pathways that mediate cell behavior. Here, they are distributed to establish functionally distinct regions that compose perineuronal nets surrounding the cell soma and proximal dendrites, interstitial matrices between neural cells, and the basement membrane between blood vessels and neural cells.^111,112^ While synaptic formation and stability rely on the perineuronal net and its dynamic composition of hyaluronan, tenascin R, and proteoglycans, the neural interstitial matrix additionally entails collagens for structural support and growth promoting glycoproteins (e.g., laminin and fibronectin). The basement membrane acts as a barrier between cell types (e.g., blood-brain barrier), and its components include collagen, laminin complexes, proteoglycans, and fibronectin.^112–114^

The heterogenic and spatial composition of the native ECM and thus mechanical and biochemical cues continuously change during the development of the brain and contribute to neural differentiation and later neural maturation;^115^ for example, the structural stability provided by collagens, laminin, and integrin proteins is favorable to neuritogenesis, cell adhesion, and migration.^114^ As such, an alteration in the regional molecular composition of the ECM can change cell fate and the subsequent ratio of excitatory and inhibitory synapses.^116^ In addition, the ratio and distribution of proteogylcans, macromolecules with bound glycosaminoglycans (GAGs), affects the regional mechanical modulus through the negatively charged GAGs attracting hydrogen and causing the interstitial area to swell.^114^ These ECM proteins bind to integrins, which subsequently affect cytoskeletal organization and the focal adhesion complex associated signaling cascades. Both wettability and charge are properties that additionally determine cell adhesion and neurite alignment.^117,118^ Nevertheless, at this stage, the understanding of the exact ECM composition in different developmental stages of the brain and its subregions is limited. However, particularly for tissue engineering, it is important to recognize the dynamic nature of ECM excretion and effects of its molecular composition on cell behavior, such as synaptic plasticity, cell organization, maturation, and network activity.^110,115,119^ The scaffolding itself provides structural stability necessary for cells to adhere to and use as a migratory guide during cellular organization.^120^ Manipulation of the structure is, therefore, possible both at the level of biomaterial composition (material fibers) and implemented bioprinting techniques (e.g., SLA). As with mechanical forces applied during printing, the mechanosensitive reaction of cells to extracellular topographical cues plays an additional role in activating signaling cascades involved in cell fate determination.^46,61,63^ The extent of the effect of a change in ECM makeup is immediately apparent through pathophysiological examples of the altered interstitial matrix composition supporting glioma growth^121^ or traumatic injury induced glial scarring inhibiting axonal regrowth.^122^ Thus, translation of this dynamic extracellular environment ideally requires upholding of the intrinsic cellular production of the ECM to allow for cell-mediated remodeling over time. This is subject to a controllable, temporary (biodegradable), and biocompatible material containing developmentally relevant molecular components (proteins and proteoglycans) and/or facilitating their delivery within the interstitial space through tissue culture media.^64^

In recognizing that there is an interplay between biochemical and mechanical properties (e.g., elastic modulus) *in vivo,* the latter's effect on cell behavior requires further investigation.^63^ Although exact mapping of mechanical properties remains limited,^123^ topographical evaluation of temporospatial variability of mechanical stiffness further highlights the heterogeneous nature of *in vivo* brain tissue and the effect of regional gradients. For example, the soft ECM that initiates ectoderm differentiation and neurogenesis becomes slightly stiffer with development and through increased myelination, favoring gliogenesis in adulthood.^77,124^ Thus, the extracellular microenvironment is part of an interactive feedback loop of cell-ECM communication, where physical forces alone do not elicit a fixed response.^61^ Interestingly, research suggests that the mechanical environment's influence on cell fate commitment is time-dependant.^125^ Rammensee *et al.* found that the mechanosensitivity of NSCs is most prominent during the initial 12–36 hours of differentiation.^125^ This is consistent with research demonstrating that early intrinsic mechanical adaptation to mechanosensitive properties by MSCs is predictive of long-term lineage specification;^126^ subjecting cells to varying mechanical stiffness outside of this time frame did not clearly impact cell fate commitment, suggestive of “mechanical memory.”^125,127^ In contrast, Lee *et al.* were able to reverse the cell fate of MSCs cultured on stiff and soft substrates, when transferring to opposing substrates after 10 days. However, their findings also illustrate that biophysical changes more readily affected early (Beta-III tubulin) rather than later neural markers (MAP2), indicating a certain degree or irreversibility.^128^ Although sensory perception is dependent on molecular components of individual cells and cell subtypes, preliminary extrapolation to iPSCs assuming a potential temporal time window is reasonable. In addition, given the counteracting/balancing effect of cell-cell adaptation to mechanical cues,^74^ it would be beneficial for further investigation to include variations of cell density in a 3D environment to better determine the extent of significance given to mechanical modulus on cell fate *in vivo*.

Further to this, inter and intraregional mechanical gradients affect not only cell fate determination but also cell migration, neurite extension, and outgrowth direction, behaviors that are also guided by fiber alignment.^129,130^ Stukel *et al.* reported that decreased material stiffness (0.1–0.8 kPa) is associated with the increased neurite length compared to the stiffer material (4.2–7.9 kPa), although neurite alignment increases with stiffness.^117,131^

Overall, neural tissue engineering requires the printed material to facilitate stem or progenitor cell survival, proliferation, and differentiation to form functional neuronal networks and supporting cells (e.g., neuroglia).^9,20,80,132^ Thus, in order to construct a scaffold with properties that mirror the ECM in human brain tissue, the physicochemical characteristics of the bioink must be considered on the basis of being biocompatible and ideally modifiable with optimal mechanical and biophysical properties (such as elastic modulus and biodegradability) afforded over time.^94^ The importance of this is highlighted by the same mechanical stimulus being able to elicit a different response as a result of a temporospatial dependent change of the biochemical or micromechanical environment^73^ and is additionally dependent on intercellular connectivity and cell type.^63,129^ Taken together with the need for favorable shear-thinning and gelation kinetics and compatibility with methods for down-stream analyses such as microscopic visualization, there is a need to commit considerable time and effort to identifying optimal cell-material combinations for effective tissue printing and modeling.^11,133^ Notwithstanding, there are recognized substitutes for the ECM of neural tissue and routinely used for bioprinting, with polymeric materials used to form hydrogels favored above all others. Among other important properties, hydrogels are water-rich and nontoxic and have physical and chemical properties that make them conducive to cell growth inclusive of neural cell support and able to approximate the mechanical properties of the brain.^134^ In particular, softer or weak hydrogels are able to replicate the elastic modulus of neural tissue (0.1–2 kPa), which is comparatively low compared to other tissues.^62^

#### 1. Hydrogels

Hydrogels include natural polymers such as collagen, gelatin, fibrin, agarose, and alginate, in addition to manufactured semisynthetic polymers such as gelatin methacryloyl (GelMA).^134^ Natural hydrogels can be subdivided into decellularized, protein- and polysaccharide-based matrices. The biocompatibility, biodegradability, and high water retention of hydrogels permit cellular encapsulation and provide an adjustable environment to act as a surrogate of the ECM in soft neural tissue. Being modifiable, hydrogel polymers can be blended to alter their characteristics, including rheological properties, and bioactive materials can be incorporated into synthetic polymers. This also allows for the production of hydrogels with a tunable stiffness gradient,^135^ likely useful for neural tissue engineering, particularly once a better understanding of regional gradients within the CNS has been established. More specifically, the biophysical parameters of the polymers may vary the amount of material swelling (water retention), printability, shape fidelity, the elastic modulus, porosity, and rates of degradation and are sensitive to external environmental conditions.^94,134,136,137^ Thorough characterization of biomaterial properties is, therefore, necessary to construct a reproducible model. In terms of the induction of neural cells in iPSC-laden scaffolds, a porous and soft tissue environment is important to enable nutrient flux, support neurite outgrowth, cell migration, network formation, and intrinsic functionality.

Hydrogels consist of hydrophilic polymer chains that can undergo gelation (copolymerisation), through noncovalent (physical) or covalent (chemical) interactions. The method of gelation is dependent on the composed hydrogel, whereby physical cross-linking is temperature sensitive (i.e., for cryogelation), and its chemical counterpart can be achieved through ionic cross-linking (e.g., for alginate-based constructs) or the addition of photosensitive cross-linking agents (e.g., for GelMA-based material) (Table II).^134,136^ Notably, the duration of cross-linking and the type and concentration of ionic crosslinker affect the mechanical properties through differences in spatial distribution throughout the material, impacting mechanical strength by means of material swelling and degradation.^138–140^ Gelation methods are generally limited by their impact on cell survival through cytotoxic components and processes, in addition to bioprinting dependent capabilities whereby the duration of exposure to the light source can modify stiffness.^141^ Thermosensitive biomaterials provide an alternative to the addition of cytotoxic cross-linking agents,^142^ although the gelation temperature may impact scaffold stability if the material state is reversible. In addition, the gelation temperature range required for solidification may impact cell viability and behavior if appreciably above or below 37 °C.^143,144^ Further to this, additional cross-linking agents may also be utilized within the bioink for increased structural stability despite a material component possessing thermosensitive gelation properties.

**TABLE II.** General characteristics of hydrogels and additives previously used in 3D bioprinting of human iPSCs and neural derivatives.

Material	Origin	Characteristics	Disadvantages	References
Agarose	Natural	Thermosensitive gelation (32 °C)	Thermosensitive (40 °C melt)	20,80,137,142
		Promotes cell proliferation and matrix production		
		Supports cell adhesion		
		Modifiable viscosity		
		Supports print-shape fidelity		
		Nonimmunogenic		
		Biodegradable		
Alginate (PS)^b^	Natural	Ionic cross-linking capability	Long-term reduction of mechanical stability	20,80,82,83,, 85,86,146,79
		Structural similarity to the EC,	Temperature/pH sensitive	
		Nonimmunogenic	High batch variability	
		Modifiable mechanical stiffness		
		Modifiable for cell adhesion		
		Low toxicity		
		Biodegradable		
(Carboxymethyl)-Chitosan(PS)^b^	Natural	Ionic/covalent cross-linking agent (supports mechanical stability)	Temperature/pH sensitive, mechanical properties	20,80,147,148
		Controlled degradability		
		Antibacterial properties		
		Conductive to cell survival		
		Promotes cell adhesion		
		Allows modification of porosity		
		Low inflammatory response		
		High moisture retention		
		Low toxicity		
		Biodegradable		
GelMA^a^	Semi-synthetic	Photo-crosslinkable	Batch variability,^c^ cytotoxic cross-linking component	87,149
		Versatile		
		Supports cell adhesion		
		Control of mechanical properties		
		Gelatin derived properties		
		Modifiable scaffold stability		
		Biocompatible		
		Optically transparent		
		Degradable		
Gelatin (P)^b^	Natural	Thermosensitive gelation (<25 °C)	Gelation temperature < 25 °C	83,87,94,140
		Enhances cell adhesion		
		Stable at high temperature and a wide range of pH values		
		Modifiable mechanical stiffness		
		Modifiable		
		arginine-glycine-aspartic acid (RGD) promotes cell adhesion		
		Biodegradable		
Matrigel	Natural	Thermosensitive gelation (24–37 °C)	Mouse tumor derived	79,150
		Supports neurite outgrowth	High batch variability	
		Scaffold stability	Poor control of mechanical properties	
		Modifiable modulus		
		Biodegradable		
Polyurethane (PU)	Synthetic	Thermosensitive gelation (37 °C)	Neural network compatibility unknown	60,84
		High water content		
		Elastic properties		
		Hydrogel combination possible		
		Biocompatible		
		Temperature sensitive crosslinker		
		Modifiable modulus		
		Biodegradable		

Additives	Origin	Characteristics	Disadvantages	References
Fibrin (P)^b^	Natural	Fiber network	Lack of mechanical stability	82,85,86,151
		Growth supporting scaffold		
		Promotes cell adhesion		
		Biodegradable		
Genipin	Natural	Crosslinking reagent (fibrin/chitosan)		82,85,86,151
		Scaffold stability		
		Promotes neurite outgrowth		
Thrombin	Natural	Fibrin polymerization agent		82,85,86,152
		Control fibril and pore size		

^a^ GelMA = Gelatin methacrylate.

^b^ (P) = protein based natural hydrogel, (PS) = polysaccharide based natural hydrogel, and (GAG) = glycoasaminoglycans (contains functional groups that bridge and link proteins to form the ECM network and provides viscoelasticity to scaffold).

^c^ Although high commercial batch variability, high control and reproducibility are possible.

Despite their batch-to-batch variation, natural hydrogels display biocompatibility, biodegradability, and bioactive and noncytotoxic properties that are desirable for cellular encapsulation and modeling.^11,142^ Protein-based hydrogels in particular occur naturally in the ECM, providing a suitable basis for a cell-laden bioink. Creating the specific microenvironment necessary for targeted cell types with natural hydrogels can be challenging, as unspecified batch variations of the protein content can alter the experimental outcome.^11^ Furthermore, Kothapalli and Kamm demonstrated that despite biocompatibility and robust neuronal outgrowth in two different natural hydrogel matrices (Collagen-I and Matrigel), addition of the same biomolecules [i.e., retinoic acid (RA) and sonic hedgehog (Shh)] can guide neuronal differentiation into two different cell types (motor neurons and dopaminergic neurons) depending on the culture medium used.^145^

Natural and synthetic polymers may be combined to increase stability and reproducibility of a hydrogel construct, in addition to enabling better control over mechanical and physical properties. Synthetic hydrogels have the benefit of being well defined and mostly support the structural aspect of scaffold construction by enabling precise adjustment of mechanical properties. Hydrogel composites offer greater scope to modify bioink viscosity and gelation to be compatible with the bioprinting procedure and formation of a stable scaffold.^140^ Bioink viscosity is not only dictated by the biomaterial components but also influenced by cell density.^81,103^ Thus, in order to achieve a 3D construct mimicking native brain tissue, cell density necessary for neural network formation must be weighed against a proportional increase in cell-laden bioink viscosity affecting the bioprinting procedure.

##### a. Hydrogels for printing human stem cells

3D bioprinting human iPSCs, neural progenitor cells derived from iPSCs, or native neural stem or progenitor cells has, thus, far principally employed natural hydrogels to construct cell-laden scaffolds (Table II), with the exception of GelMA and synthetic hydrogel polyurethane (PU). The majority of biomaterial combinations contain the anionic polymer alginate, which can be ionically crosslinked to form a scaffold of low (∼0.010 kPa) to high (∼4 kPa) mechanical stiffness, depending on the concentrations of alginate and cationic components (e.g., chitosan).^147,153,154^ Consistent with the mechanical properties of a biomaterial influencing cellular properties, material with lower mechanical stiffness (0.1–1 kPa), correlating with the modulus of the native ECM in brain tissue (0.5–1 kPa), supports neuronal adhesion and differentiation. In contrast, the adherence of glial cells and subsequent survival is associated with stiffer gels and accompanied by a reduction in neuronal differentiation and neurite branching.^126,142,155–157^ In the developing brain, mechanical stiffness increases with age, supporting gliogenesis over neurogenesis at later developmental stages.^77,158^ Hydrogels with lower viscosity are, therefore, necessary for neural tissue modeling and importantly are also bioprintable with the aforementioned techniques. Maintaining shape fidelity, however, presents the largest challenge in low viscosity materials. Despite its importance, the mechanical modulus is seldom considered for previously reported 3D bioprinted neural models (Table I).

Hsieh *et al.* created a thermo-responsive synthetic hydrogel (PU) to circumvent the need for introducing potentially cytotoxic components of chemical cross-linking agents. The gel also affords a modifiable, more reproducible, and consistent scaffold unaffected by batch-to-batch variation emblematic of natural hydrogels^60^ and supports NSC proliferation and differentiation to mature neurons. While Ho *et al.* similarly describe neural induction of PU encapsulated human iPSCs, neither reported neurite outgrowth or cell functionality.^84,139^ Zhu *et al.* constructed a semi-synthetic GelMA scaffold with integrated graphene nanoparticles, which supported both neural differentiation and neurite outgrowth.^108^ Also, Sharma *et al.* recently described the addition of microspheres containing guggulsterone for controlled drug release and NPC differentiation to dopaminergic neurons.^82^ Taken together, the studies highlight a diversity of materials and mechanisms that can be utilized to promote and guide neural differentiation within hydrogels and associated bioinks employed for printing.

Both natural and synthetic hydrogels used for 3D bioprinting human iPSCs promote various ECM-supported cellular activities (Table II), such as the formation of fiber networks by fibrin or the enhancement of cell attachment with the addition of gelatin.^136,151,152^ Nevertheless, many natural ECM components remain to be incorporated into a printable biomaterial with a view to providing better support and/or functional benefits. Collagen, for example, is a common protein in the CNS and has been used as an injectable scaffold, providing structural support for brain tissue repair.^159^ Oyama *et al.* also reported using a collagen scaffold for long-term culture of iPSC-derived neuronal cell aggregates with strong cellular adherence.^160^ Still, standardizing biomaterials for printing and building 3D cellular constructs has a long way to go, as assembling the components of the ECM is challenging. This includes determining the appropriate ratio of numerous known and unknown factors and associated properties toward artificially recreating the microenvironment.

### C. 3D bioprinted neural models

The current research of 3D bioprinted neural models derived from human iPSCs is limited, with published studies varying significantly in terms of methods employed and findings (Table I).

**FIG. 3.**
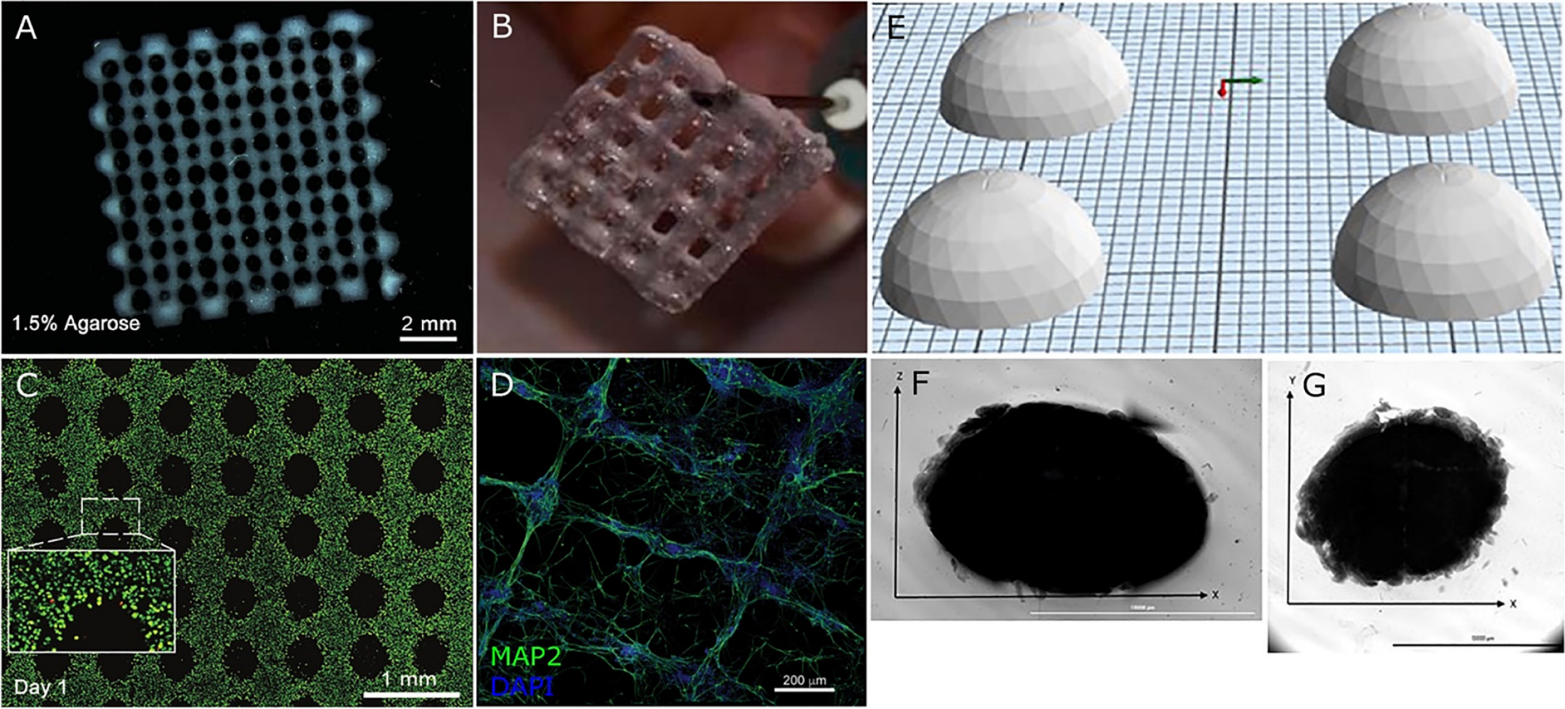
Selected examples of 3D bioprinted neural scaffolds. (a and b) Cross-hatch, macroporous structures containing (a) NSCs in an Al/Ag/CMC-based hydrogel.^20^ (b) Neuroblastoma cells in an Al/Gel hydrogel.^20,83^ (c) Live-dead (green/red) cell staining within a cross-hatch structure of an Al/Ag/CMC-based hydrogel containing iPSCs.^80^ (d) Neuronal alignment within a cross-hatch scaffold of a Matrigel/Al hydrogel.^79^ (e and f) A printed dome-like structure of an Al/chitosan-based hydrogel,^82^ with (e) showing CAD images and (f and g) showing a printed, NPC-laden dome structure. Reproduced with requisite permissions from Gu *et al. Adv Healthcare Mater.* **5**, 12 (2016) and *Ibid.* **6**, 17 (2017). Copyright 2016 and 2017 Wiley-VCH, respectively, and Sharma *et al. Front. Bioeng. Biotechnol.* **8** (2020), Fantini *et al. Cells* **8**, 8 (2019), and Salaris *et al. J. Clin. Med.*, **8**, 10 (2019). Copyright 2020, licensed under a Creative Commons Attribution (CCBY) License. Al = alginate, Ag = agarose, CMC = carboxymethyl-chitosan, and Gel = gelatin.

In each case of 3D printed neural models, the cell viability within printed iPSC constructs was 50%–100% post-printing, with a tendency toward further cell proliferation in the days following. The lowest viability appears to correlate with synthetic and semisynthetic biomaterials, although probably due in part to the use of printing shear stress for transfection.^84,87^ Synthetic materials aside, and to reiterate, extrusion printing are associated with both lower and high viability scaffolds, indicative of the effects of variable and more or less optimal shear stress on cell survival. A prolonged process (>15 min) of dehydration of cell-laden biomaterial can also significantly reduce cell viability.^87^ The comparative analysis of 3D printing iPSCs and NSCs by Fantini *et al.* further demonstrated that cell viability did not vary between each cell line printed within the same material (Gelatin/Sodium Alginate).^83^ Repeatability tests within this study confirmed temperature (25 °C) and concentration (6% sodium alginate and 4% gelatin) dependent high scaffold fidelity and reproducibility through extrusion printing [Fig. 3(b)]. However, low cell density and limited methods that aim to investigate neural connectivity and neurite extensions impede critical evaluation of this biomaterial as an adequate scaffold for neural tissue modeling.^83^ On the other hand, Joung *et al.* (2018) reported that gelatin and fibrin hydrogel were not able to support NPC viability over a 4-day observation period, although the viability of fibroblasts remained stable. The culture medium was supportive of neural cells, suggesting inadequate mechanical properties of the hydrogel. It is reasonable to assume that the gel's high mechanical stiffness was supportive of fibroblast growth but not conducive to maintenance of neural cells. Notwithstanding, stem cell viability and inherent self-renewal illustrate the ability of scaffolds to support 3D cell aggregates and colonies, as seen in Gu *et al.*, without the complication of a necrotic core observed in traditional 2D and 3D stem cell cultures and derivative 3D organoids.^21,161^ Notwithstanding the differences between reports, one research group demonstrated the reproducibility and versatility of their fibrin and alginate-based bioink and printing technique by initially showing biocompatibility with iPSCs followed by 3D printing of neural progenitor cells (NPCs) and neural aggregates (NA).^82,85,86^ Sharma *et al.* added to this earlier work by incorporating microspheres to promote controlled drug release toward neural differentiation of the NPCs, without compromising cell viability.^82^ Although the addition of guggulsterone led to more mature neural differentiation than addition of an unloaded microsphere, biomarker imaging demonstrated limited neural networking after 30 days.^82^ This is despite previous modeling with the same bioink and NPC differentiation involving neural network formation.^86^ Microfluidic printing of NAs in this fibrin/alginate based bioink does not show neural connectivity beyond the individual aggregates after 36 days.^85^ Unfortunately, neither of the above studies specify cell density used, which may influence network formation. Surprisingly, despite the extended culture length (30–36 days), there is a general shortfall of studies addressing neural maturation and functionality. Interestingly, in contrast to the studies for printing NPCs, biomarker staining of the NAs was negative for the glial marker GFAP, suggesting a homogenous neuronal population.^85^ This is consistent with previously mentioned research highlighting the effect on cell connectivity with behavioral outcomes to environmental cues.^74^ Therefore, increased cell-cell communication (within NAs) may influence cell fate and be associated with density-dependent changes/interplay between biophysical and biochemical cues of the cell-created microenvironment. The significance of the cell density is further apparent from Joung *et al.*, demonstrating that higher cell density was associated with a 100% increase in the survival rate upon exposure to untenable environmental cues.^87^

Interestingly, all studies report the development of a 3D bioprinted scaffold in which neural differentiation was induced and/or maintained, with the majority initiating induction post-printing (Table III), supporting self-organization akin to native tissue. Also notable, the high variance in the duration of cell support by scaffolds of different studies to some extent reflects the different paradigms for end-point analysis (Tables III and IV). Moreover, the scaffold designs implemented were either cross-hatch macroporous constructs or dome-shaped structures (Fig. 3). The cross-hatch structures show high shape-fidelity, whereas the proportions of the 3D printed dome-shaped material appear inconsistent. For a reproducible 3D model, consistency inclusive of shape-fidelity is vital and its lack thereof compromises the ability to accurately/decisively compare *in vitro* models. In addition, although hydrogels create a microporous environment for cell growth, incorporation of regularly spaced pores or lumen within a printed construct enables increased and uniform penetrance of nutrients throughout the model, resembling vasculature. This prevents nutrient access-related hypoxia and necrosis of cells within the 3D core, as observed in larger spheroid and organoid models. More specifically, the overall maintenance of the cell-laden neural scaffolds ranges from 4 to 70 days, which does not allow a direct comparison of long-term culture and the effect of scaffold biodegradation on the cells.

**TABLE III.** Biomaterial specifications and neural induction of 3D bioprinted human iPSCs for neural models. ^*^Cross-linking agents. ^**^Reprograming from fibroblasts during the extrusion process. The transfection rate is 15.6%. ^1^Neural induction media added. ^2^Neural differentiation media added. ^L^Research from the same laboratory group. ^AP^ = initiation of differentiation after printing and ^BP^ = initiation of differentiation before printing.

Reference	Scaffold material	Concentration	Initiation of differentiation	Time of 3D cell culture	Cell density	Neurite extensions	Functional
82^L^	Fibrinogen	20 mg/mL	Day 0^AP^	30 Days	N/A	Medium	N/A
	Alginate	5 mg/mL					
	Genipin	0.3 mg/mL					
	Chitosan^*^	0.75 mg/mL					
	Calcium chloride^*^	20 mg/mL					
	Thrombin^*^	1.7 U/mL					
83	Alginate	4 %, 6 %	Day 7^AP^	30 Days	5 × 10^4^/mL iPSCs	Low	N/A
	Gelatin	4 %					
					2 × 10^6^/mL NSCs		
85^L^	Fibrin	20mg/mL	Day 17^AP^	36 Days	10.000 per NA	Within individual NAs	N/A
	Alginate	5 mg/mL					
	Genipin	0.3 mg/mL					
	Chitosan^*^	0.75 mg/mL					
	Calcium chloride^*^	20 mg/mL					
	Thrombin^*^	10 U/mL					
79	Alginate	2 %	4w^BP^	70 Days	N/A	High	Yes
	Matrigel	50 %					
86^L^	Fibrin	20 mg/mL	Day 1^AP^	30 Days	N/A	High	N/A
	Alginate	5 mg/mL					
	Genipin	0.3 mg/mL					
	Chitosan^*^	0.75 mg/mL					
	Calcium chloride^*^	20 mg/mL					
	Thrombin^*^	1.7 U/mL					
87	GelMA	7.5%		4 Days	1 × 10^7^/mL	Low	N/A
	Gelatin	7.5%					
	Fibrinogen	10 mg/mL					
	Matrigel	50%	18 Days^BP^	14 Days	1 × 10^7^/mL	High	Yes
84^**^	Polyurethane	25–30%	Day 2^AP^	14 Days	1 × 10^6^/mL	N/A	N/A
80	Alginate	5%	^1^Day 3^AP^	40 Days	8 × 10^7^/mL	High	Yes
	Agarose	1.5%	^2^Day 20^AP^				
	Carboxymethyl-chitosan	5%					
20	Alginate	5%	Day 5^AP^	31 Days	1 × 10^7^/mL	High	Yes
	Agarose	1.5%					
	Carboxymethyl-chitosan	5%					

**TABLE IV.** Summary of analytical methods used to determine neural differentiation: listed in alphabetical order.

References	IHC	RT-qPCR	Other
Sharma *et al.*^82^	FoxA2, TH, TUJ1	LMX1B, NR4A2, PAX6, TH, TUBB3	Flow cytometry: GFAP, O4, TH, TUJI
Abelseth *et al.*^85^	GFAP, TUJI1		
Fantini *et al.*^83^	Nestin, PAX6, Sox2	Nestin, PAX6, SOX1, SOX2	
Salaris *et al.*^79^	GFAP, MAP2, NCAP, NeuN, PAX6, TBR1, TUJI	FOXG1, GFAP, PAX6, TBR1, TBR2	Patch clamp Calcium imaging
De la Vega *et al.*^86^	ß-tubulin-III, ChaT, GFAP		Flow cytometry: ß-tubulin III, HB9, Olig2
Joung *et al.*^87^	ß-III tubulin, NeuN, Sox10		Calcium imaging
Ho *et al.*^84^	FoxD3	ß-tubulin, FoxD3, GFAP, MAP2, Nestin, Sox10	Western Blot: ß-tubulin, GFAP, Nestin
Gu *et al.*^80^	GABA, GFAP, MAP2, Nestin, PAX6, SOX2, Synaptophysin, TUJ1	GABA, GFAP, NES, NKX2-1, OLIG2, PET1, TUBB3	Calcium imaging
Gu *et al.*^20^	GABA, GAD, GFAP, KI67, Nestin, OLIGO2, SOX2, Synaptophysin, TUJ1	GABA, GFAP, MYST, NKX2.1, OLIGO2, PET1, SRT, SYP, TUJ1, VGLT	Calcium imaging

Comparative material analysis, if possible, is limited by unknown batch-to-batch variation between studies, in addition to the limited and inconsistent information on cell density affecting material properties, such as viscosity. For seven of nine studies, alginate is a component of the bioink (Table I), although a different batch of alginate from even the same manufacturer may present with significantly different properties, affecting the concentration required for suitable bioink preparation, printing, and gelation.^94^ One variable factor is alginate's molecular weight, which can alter the material's rate of biodegradation, mechanical moduli, and correlated viscosity.^87,146^ This also applies to other biomaterial components (e.g., chitosan) and their biodegradability, which are also affected by their molecular weight.^148^ Another source of variation relates to the composition of alginate's copolymers, L-guluronic acid (G monomer) and D-mannuronic acid (M monomer). As a naturally occurring seaweed derived substrate, alginate's relative proportion and distribution of M and G monomers differ from one batch to another through the influence of environmental factors during its life cycle. More permeable alginates are thereby linked to a higher M/G ratio and less permeable alginates to a lower ratio. This change in permeability allows variable influx of ionic cross-linking agents, thus altering alginate's physicochemical properties.^146,162,163^ Importantly, the same can be said for commonly used tumor-derived Matrigel with batch dependent component variations, as well as GelMA, whereby during production, the modification of the gelatin synthesis with methacrylic anhydride (MAA) affects the materials' biophysical properties.^79,87,149,150,164^ These differences are often not indicated, and although variations are unavoidable with some materials, rheological methods including steady shear and small-amplitude oscillation can be used to assess batch variability.^165^ In any event, the materials' properties are affected, such as viscosity. Therefore, comparing between studies should take these batch variabilities, as well as potential confounders such as the ranges of material concentrations employed to support neural differentiation, whether used alone or in combination with other biomaterials, into account (Tables II and III). The materials considered by the studies are biodegradable, although only one reported biodegradation of printed scaffolds over time (30 days) with replacement by the cell-secreted ECM, supported by cellular attachment to the cell culture well. Further evidence to support this claim is, however, not provided.^86^ Abelseth *et al.* employed the same bioink and reported minimal visible degradation and did not consider biomaterial replacement with the intracellularly produced ECM.^85^ The altered mechanical properties of another alginate-based bioink have been reported, including a reduction of the initial mechanical stiffness of 7.5 kPa following cross-linking of the material to 0.8 kPa within 10 days post-printing.^20^ This increased stiffness correlates with the time period in which differentiation was initiated and subsequently the temporal window of increased mechanosensitivity of cells, influencing fate determination.^125^ Nevertheless, despite scaffold maintenance for 40 days, no further investigation of material properties was undertaken thereafter to assess material stability or replacement by the ECM.^20^

Table IV highlights the analytical methods used for fundamental detection of neural differentiation, including immunophenotyping and transcriptional analysis, toward the assessment of the particular cell type and functionality. Assessments of neurite outgrowth and network formation have not been consistently undertaken, and only four studies assessed the functionality of the neuronal cells within the 3D scaffolds.^20,79,80,87^ Salaris *et al.* verified the function of immature neuronal cells 7 days post-printing, and Gu *et al.* demonstrated functional maturing neurons after 30 days in their printed constructs.^79,80^ Joung *et al.* recorded spontaneous calcium flux in neurons within Matrigel 14 days post-printing, although NPC/OPC fate had been induced for a total of 18 days prior to printing.^87^ The latter study, however, did not provide further biomarker analysis of neural characteristics and maturity. Overall, these studies illustrate the ability to form functional neurons via 3D bioprinting with materials exhibiting different properties. Salaris *et al.* illustrated higher cell adhesion with extensive neural network formation and continued maturation for up to 70 days after printing, in which neurites track along struts of the scaffold.^79^ Gu *et al.* on the other hand also created embryoid bodies (EB) prior to differentiation, with iPSCs proliferating to form 3D cell aggregates suspended in the printed matrix. Although offering the potential to self-assemble within the construct, similar to that of organoids, directed differentiation to neural lineage resulted in GABAergic and serotonergic neuronal subtypes and putative oligodendrocytes.^80^ In addition, consistent with the presence of GABAergic neurons, neuronal function (evidenced by live-cell calcium flux imaging) was affected by treatment with GABA receptor-A antagonist bicuculline. Functionality was also supported by live-cell imaging of migrating cells within constructs.

Further research into optimal biomaterials is necessary and should consider neural differentiation spatiotemporally and maturation, toward better ascertaining tissue developmental potential and clinical relevance of 3D bioprinted models. Nevertheless, natural hydrogel alginate-based bioinks have been shown to provide a structurally stable 3D printable scaffold with modifiable physical properties and biodegradability that supports neural tissue formation. The bioprinting process appears to have a less significant effect on the cellular outcome than the biomaterials encapsulating the cells, although it influences the selection criteria and concentration of the materials used. Again, batch-to-batch variation remains a challenge as natural hydrogels provide bioactive components that, although intended to mimic the native ECM, vary in terms of ratios and functionality. Therefore, without well-defined molecular properties of natural hydrogels, a consistent and reproducible scaffold and differentiation protocol is difficult to achieve often requiring continuous fine-tuning. For this reason, more stream-lined and detailed experimental analysis is required, for biomaterials' characterization and cellular effects, toward standardized and reproducible 3D neural tissue models.

Consideration must also be given to directing differentiation into specific neuronal cell types representative of *in vivo* human neural tissue with regional specificity. This could be achieved through the use of hydrogels with a tunable stiffness gradient^135,166^ or targeted release of biochemical cues.^82^ This includes recapitulating the desired native tissue type, ratio, and distribution of glial cells and neurons (1–4:1).^167^ In regard to biomaterials, given the ability to adjust the concentration of polymers such as alginate to modify gel stiffness, a scaffold containing an alginate gradient may be constructible with a printing technique that facilitates the distribution of multiple cell-laden bioinks on a layer-by-layer basis or via other configurations such as core-shell printing through co-axial extrusion.^95,166,168^ Similarly, the microfluidic variant of extrusion printing allows modification of the flow rate of the separate material components. This enables dynamic adjustment of material (e.g., cross-linking agent) concentrations throughout the printing process to create mechanical stiffness gradients within the printed construct and ensure shape fidelity.^87,96^ In addition to reduced shear stress acting on cells, this controlled distribution of varying material types and concentrations may enable a more precise representation of native cellular arrangements or synthesis of optimally engineered synthetic constructs that are more fit-for-purpose than merely casting cells within a hydrogel, without spatial control.^166^ Although (microfluidic) extrusion printing does not allow the precision single-cell spatial distribution (and resolution) that inkjet and laser-assisted printing provides, a controlled mechanical gradient with a homogenously high cell density is arguably more beneficial for mimicking *in vivo* tissue, particularly in post-printing induced differentiation of iPSCs that undergo migration and self-organization, which in turn can be guided. However, a more in-depth understanding of topographical stiffness variations within particular brain regions is required.^123^ In contrast, printed constructs for cell replacement therapies and clinical transplantation would benefit from differentiation prior to printing to ensure a more controlled and homogenous outcome. In this case, exact structural guidance of neurons and axonal projections might be required, for which high resolution bioprinting techniques with the ability to create topographical guidance cues (e.g., SLA electrospinning)^109^ could be advantageous.

Whatever the case, with the ultimate objective of creating printed constructs able to support physiologically active and interacting neural cells, tailoring mechanical performance, degradation behavior, and biocompatibility for inter-cellular electrical signaling is key. These studies (Tables I and III) provide a foundation for proof-of-concept 3D bioprinted modeling of neural tissue, although an increased emphasis on the construction and thorough analysis of more mature and functional neural networks is required. As fully matured neural networks exhibit complex synchronous firing in addition to spontaneous neural firing, the exclusive presence of the latter as observed for the above-mentioned extended culture periods is indicative of still ongoing neural maturation. Extending culture periods over a matter of months and years to achieve adequate recapitulation of mature *in vivo* neural tissue is not only impractical but also insufficient for possible disease modeling toward individualized treatment approaches. Previously stated developmental changes associated with the ECM of *in vivo* neural tissue are associated with cellular maturation and should be used as a reference to guide future bioengineering toward establishing the engineered scaffold's temporal replacement with the intracellularly produced ECM. This requires assessing the rate of biomaterial degradability and use of analytical techniques (e.g., immunohistochemical staining) to examine the presence of cell-secreted matrix components. While intrinsic regulation is important, more recently exogenous electrical stimulation (ES) has been applied as an addendum to printing to augment tissue fabrication by facilitating both neural maturation and ECM production.^46,169^ Additionally, electrical stimulation has been investigated as a tool to regulate cell determination and organization.^46,170^ As such, this review considers in the next section ES applied to neural tissue models, with a view toward combining both ES and 3D bioprinting to create enhanced and even more relevant and reproducible *in vitro* neural tissue analogs.

## III. ELECTRICAL STIMULATION OF HUMAN STEM CELLS FOR ADVANCED NEURAL TISSUES

Application of ES for *in vitro* neural modeling has addressed some of the shortfalls described for conventional 3D modeling, which apply to 3D bioprinted neural models. To reiterate, mechanosensitive responses to the cellular environment can enhance maturation and alter cell fate, although mechanically and chemically induced cytoskeletal reorganization and downstream cellular responses do not represent the entire scope of cell-influencing cues.

Neurons in native tissue are electrically active and communicate via electrical and chemical signals. The electrical signals transmit information from one neuron to the other to form neural networks.^171^ This intercellular communication is regulated by the electroconductive property of the neural microenvironment, inclusive of the ECM.^172^ *In vitro* research has confirmed that culturing cells on the electroactive material enhances the expression of the neural differentiation and maturation marker MAP2, beyond nanotopographic and thus mechanosensory induced changes.^46^ As is the case for cellular mechanosensitivity, different types of neuronal cells are functionally distinct and have their own unique intrinsic electrophysiological properties. These properties are defined by the cell morphology (dendritic length and diameters) and passive and active membrane characteristics. This underlies the inherent electrical activity that contributes to intercellular communication in neural networks, inclusive of synaptic current from excitatory or inhibitory synapses.^173–175^ For example, the vast interconnected neural network of the brain undergoes constant firing of 40 Hz gamma oscillations while in the conscious state. Direct and indirectly correlated downstream events stemming from these gamma oscillations in the cortico-thalamo-cortical network contribute to the cognitive function, in line with the strengthening of synaptic connections.^173^ To the same effect, Yokio *et al.* observed that synaptic weakening of dopaminergic neurons in the ventral tegmental area (VTA) was induced when exposing human iPSC derived neurons to a 1 Hz low-frequency stimulation (LFS), mimicking non-rapid eye movement (REM) sleep.^176^

This highlights the influence of inherent properties and external variance of properties on a cell's electroresponse and altered cellular output (e.g., oscillation frequency and cell behavior). This can occur despite exposure to a consistent current.^173,174,177^ Both immature and mature neurons are excitable and fire spontaneously. Even at early developmental stages, this intermittent activity can be synchronized in small neuronal networks, ahead of developing more complex synchronous activity upon formation of a mature neural network.^178,179^ Glial cells, on the other hand, are not electrically active (i.e., do not fire action potentials) but are susceptible to exogenous electrical activity and stimulation. Nevertheless, electrical activity, whether through intrinsic or exogenous (artificial) stimulation, influences cell behavior such as proliferation, migration, and differentiation at all developmental stages.^180,181^ The guidance of cell behavior and fate determination through exogenous ES are, therefore, dependent on the specific cell type (e.g., pyramidal, spinous, thalamic, or cortical neuronal) to determine the required stimulation frequency, duration, and voltage.^173,182^ The benefit of ES as a method for regulating differentiation, and enhancing proliferation and migration is the precise control that can be exerted through application of predefined voltage and frequency, without the introduction of variable chemical activities, batch discrepancies, and remaining chemical residues.^182^

To achieve effective stimulation of iPSCs without compromising biocompatibility, electroactive biomaterials such as conductive polymers (CPs) and metal nanoparticles (NPs) can form or be incorporated into cellular scaffolds. Stimulation can, then, be initiated through connecting electrodes to create an electrical field (EF) or via an indirect electromagnetic field (EMF) platform through electromagnetic induction, reflective of clinical transcranial magnetic field stimulation (TMS).^182,183^ EFs generated from electrodes are currently the most common method of *in vitro* stimulation of stem cells or derivative neural cells. Implementation of a wide range of stimulation parameters comprising direct current (DC) EF, pulse-current (PC) EF, and biphasic electrical current (BEC) EF stimulation has highlighted the relevance and potential of ES for enhanced modeling of neural tissues.^72,182^

The effect of ES on human derived stem cells [NSCs, iPSCs, embryonic stem cells (ESCs), adipose stem cells (ADSCs), and bone marrow mesenchymal stem cells (BM-MSCs)] has demonstrated its role in increasing multiple behavioral and cellular properties, ranging from migration,^177,184^ neurogenesis,^185^ survival,^186–188^ differentiation^45,46,170,186,188–192^ morphological changes,^170,187,193^^,43,^^45,189,191^ maturation,^43,46,185^ and function.^43,46^ While understanding of underlying mechanisms is still limited, ES-mediated changes are ostensibly a result of EF induced modification and reorganization of the cytoskeleton and plasma membrane, with a redistribution of membrane receptors (e.g., ion channels), in addition to activation of intracellular signaling pathways, including Ca^2+^^72,182^ Application of exogenous stimulation alters the extracellular electrochemical gradient and protein absorption.^194^ An implication of this is the rearrangement of the cytoskeletal filaments, correlating with conformational alterations of the adhered plasma membrane through the potential aggregation of glycolipids, which upon accumulation form polarized rafts guiding migration.^72,195^ EF initiation of voltage-gated calcium channel (VGCC) Ca^2+^ influx and intracellular Ca^2+^ release from the endoplasmic reticulum leads to depolarization and an action potential (AP), an action associated with functionally active neurons.^171^In addition to an AP, intracellular Ca^2+^ is able to instigate phosphorylation of the cyclic adenosine monophosphate (cAMP) response element binding protein (CREB) via CaMKII activation. Ensuing CREB-mediated protein transcription (e.g., neurotrophins) influences cell survival, migration, outgrowth, differentiation, and maturation.^72,196–198^ In response, the release of neurotrophins [e.g., brain-derived neurotrophic factor (BDNF) and nerve growth factor (NGF)] initiates activation of downstream signaling pathways such as PI3K, ERK, Phospholipase C (PLC), and MAPK, which, amongst stimulation of CREB-signaling, further influence the cellular response to ES and synaptic plasticity;^46,72,197^ This is associated with a PI3K, MAPK/ERK dependent modulation in synaptophysin (SYN) and postsynaptic density protein 95 (PSD-95) protein synthesis, as well as vesicle trafficking, to promote neurite outgrowth and other CREB-dependent induction of neural differentiation and maturation markers (e.g., MAP2).^199–201^ Further synaptic plasticity associated N-methyl-D-aspartate (NMDA) receptor enhancement underlying long-term potentiation mediated by CREB-signaling, induced c-fos transcription.^72^

In addition to affecting cell behavioral signaling cascades, the ES dependent increase in neural activity additionally supports the formation of the perineuronal nets surrounding neural cells and enhances astrocytic ECM secretion.^169,172^ This ES induced replacement of the ECM is valuable to accelerate the formation of neural networks,^115^ enhance maturation, and account for developmentally dependent dynamic changes within neural tissues. Nevertheless, the variation of the EF orientation, strength, timing, and frequency is a source of modification of these cellular responses.^177,195,198^

Given interspecies variations of findings regarding ES, the adequacy of animal models for data extrapolation to human tissue must be questioned, despite the ability to draw parallels between murine and human EF studies.^177^ As an example, Kalmbach *et al.* underscored the divergence of electrical properties of mouse neurons from human neurons.^3^ Accordingly, here we only consider modeling with human derived neural cells for human biological and translational relevance.

### A. Planar/2D electrical stimulation

The low current (16 mV/mm for 1 hr) DCEF stimulation of human NSCs (derived from ESC line H9) differentiated into neurons and astrocytes reported by Feng *et al.* provides evidence for time and voltage dependent galvanotaxis toward the cathode. An increase in EF current (up to 300 mV/mm) led to an increased speed of the same direction galvanotaxis.^177^ In concordance, a mono-directional pulsed EF current of 250 mV/mm applied to human NPCs significantly increased the migration distance and was partially linked to the EF's influence on intracellular Ca^2+^ signaling.^184^ Using a biphasic waveform (0.25 mA/cm^2^, 100 us pulses), Stewart *et al.* recurrently electrically stimulated human NSCs attached to an electroactive CP polypyrrole (PPy) containing the anionic dopant dodecylbenzenesulfonate (DBS) for enhanced biocompatibility, inducing glial and predominantly neural differentiation in addition to enhanced neurite outgrowth.^45^ As with Feng *et al.*, the authors also observed galvanotaxis toward regions of higher electrical conductivity.^45,177^ This technique was later applied to human iPSCs, with the aim to produce a more patient-specific and translational platform.^189^ Despite the ability of iPSCs to give rise to all cell types of the body, as opposed to more restricted multipotent NSCs employed by Stewart *et al.*, ES induced cells of the three germ layers from the iPSCs but with the addition of neurobasal media, a clear bias toward neural induction was recorded, with neuronal over glial fate determination.^45,189^

Du *et al.* reported EF stimulation of human neural crest stem cells (NCSCs) cultured on cathodes. Correlating with ES amplification of signaling and transcription pathways, neural differentiation was enhanced compared to nonstimulated cells by applying 200 mV/mm at a stimulation frequency of 20 Hz and 100 *μ*s pulse.^186^ Another study analyzing neuronal differentiation of suspended human ADSCs when subjected to EF found that stimulation initiates neuronal differentiation and elongation without chemical induction factors. With the addition of copper containing electrodes, an overall upregulation of early (ßIII-tubulin expressing) and mature (MAP2 expressing) neuronal markers was detected.^170^ Similarly, human MSCs seeded on to the CP substrate, polyaniline (PANI), displayed neural-like filopodial extensions, and mRNA analysis revealed the expression of the neural markers Nestin and ß-III tubulin.^192^ Investigating human NSCs exposed to 1 V EFs with a stimulation frequency of 100 Hz and 10 ms pulses on CP poly(3,4-ethylenedioxythiophene) polystyrene sulfonate (PEDOT:PSS) illustrated enhanced differentiation, neurite outgrowth, and positive expression of neural markers (GFAP, ßIII-tubulin/Tuj1).^191^ Interestingly, Yang *et al.* compared the application of repeated 10 uA ES of NSCs at a low frequency (1 Hz) on a titanium (Ti)-coated nanopatterned verses flat substrate, which (similar to Pires *et al.*) enhanced neural differentiation and neurite outgrowth.^46,191^ Moreover, not only did the nanopatterned groove encourage neural alignment, in comparison to a Ti-coated flat surface substrate, but also electrophysical properties indicating functional maturation were observed in differentiated NSCs.^46^

### B. 3D electrical stimulation

Although the above findings provide insight into the beneficial effects of ES on a given cell type for planar/2D cell culture, cell effects through exposure to an electric field can differ in 3D modeling, for example, reduced global synchrony of firing.^202^ This correlates with enhanced maturation associated with the nanotopographical changes in Yang *et al.* ^46^ Furthermore, the cellular galvanotaxis of brain tumor initiating cells (BTICs) and metastatic disease causes migration toward opposing electrodes depending on the modeling dimensions.^56,203^ Further emphasizing the cellular disparity of intrinsic electrical properties, Zhang *et al.* found the opposite galvanotaxis of human iPSCs and ESCs upon exposure to an EF.^204^ This additionally highlights the need for, and importance of, a standardized 3D tissue model regarding cellular origin, mechanical and physical properties of biomaterials, and the methods applied for tissue building.

**FIG. 4.**
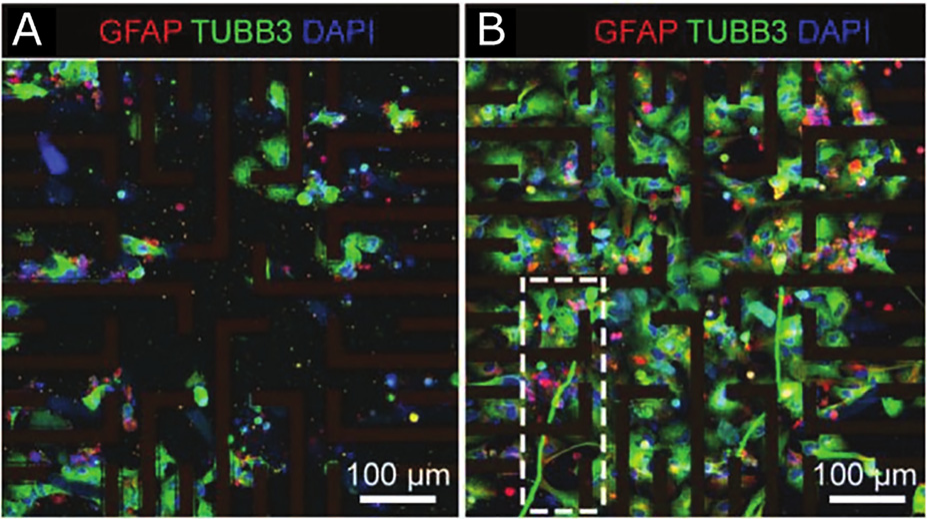
3D printable gel-encapsulated human neural cells derived from human neural stem cells on 3D conductive polymer pillar microelectrode arrays (A) without electrostimulation and (B) with electrostimulation, with the latter displaying an increased number of neural cells and connectivity.^43^ Reproduced with permission from Tomaskovic-Crook *et al.*, Advanced Healthcare Materials, **8**, 15 (2019). Copyright 2019 Wiley-VCH.

Notwithstanding the current dearth of research of ES of 3D human derived neural cell models, 3D findings appear to be consistent with 2D findings of augmented neuronal differentiation and maturation.^43,185,190^ Although Yang *et al.* demonstrated that cellular effects of ES are less significant when combined with biophysical cues (i.e., culturing in nanogrooves rather than on a flat substrate), both electroconductivity of the material and ES augment neural tissue modeling outcomes.^46^ Building on our own previous 2D work, Tomaskovic-Crook *et al.* described a printed array of 3D penetrating CP pillar electrodes used to stimulate a 3D conductive polymeric construct with encapsulated human NSCs. Stimulation in the neural induction medium induced neural differentiation and maturation, with stimulated neurons exhibiting higher colocalization of MAP2 with synaptic vesicle marker SYP, compared to unstimulated cells. Analysis of ES models subsequently supported enhanced formation and preservation of functional neural networks (Fig. 4).^43^ The recorded increase in calcium flux of the ES model correlates with amplified cellular modifications (e.g., neuritogenesis, differentiation, and maturation) related to an upregulation of previously described CREB-associated signaling cascades. Notably, the conductive biomaterial used is the same as that described by Gu *et al.* and is, therefore, 3D printable.^20^ This also suggests that despite enhanced maturation of cells being achieved on conductive constructs alone, additional ES further augments development. Similar to 2D ES, there was an increased ratio of neuronal to glial cell induction, suggesting that ES could be used to modulate the content of neurons relative to glia within engineered tissues, perhaps to emulate native tissue.^43,167^ However, dynamic changes in the *in vivo* ECM throughout development coincide with preferential gliogenesis upon mechanical stiffening of the microenvironment. This suggests that an adjustment of the neuronal to glial ratio may occur upon neural tissue maturation and subsequent ECM replacement over time, although this would require investigation of the materials replacement in 3D *in vitro* models with intercellularly produced ECM and extended culture periods. Finally, Heo *et al.* recently reported enhanced neural induction of 3D human ADSC-aggregates in electroconductive polyethylene glycol (PEG)/PEDOT:PSS microwells with ES compared to the non-ES equivalents and the use of a nonelectroconductive hydrogel, although did not record functional maturation.^190^

In addition to ES for neural tissue engineering, therapeutic ES has recently been modeled *in vitro* using Rett syndrome patient-derived human iPSC-NPCs in a 3D graphene scaffold. The constructs recapitulated the cellular disease pathology of reduced soma size, diminished dendritic branches, and decreased neural maturation. Application of EF stimulation at a frequency of 1 Hz at 10 uA (30 min/day for 3 days) increased cell soma size and MAP2 expression associated with ES induced signaling pathways for enhanced cellular maturation.^185^ This is consistent with clinical findings of therapeutic benefits posed by deep brain stimulation (DBS) and corroborates earlier 3D ES of iPSC derivatives for enhanced *in vitro* neurogenesis.

## IV. COMBINING 3D BIOPRINTING AND ELECTRICAL STIMULATION FOR ENHANCED *IN VITRO* NEURAL TISSUE MODELLING

The biochemical and biophysical cues enhancing and guiding neuritogenesis, neural differentiation, and maturation differ in electrical stimulation and 3D bioprinting. While electrical stimulation triggers cellular responses to ion influx and action potentials, 3D printing also incorporates the force-mediated mechanosensitive response to material stiffness, nanotopography, and printing induced shear stress. Unless otherwise stated, the bioprinting process is not utilized for its force-induced behavioral changes (e.g., ion influx and activation of signaling pathways) and aims to provide minimal impact on these cascades, rather serving as a tool for controlled and reproducible modeling. The material characteristics (e.g., biocompatibility) are, however, fundamental for the initial viability of the tissue model and cell behavior, including the differentiation paradigm and neuritogenesis. Material dependent biomechanical cues that interact with mechanosensitive receptors of the cells can affect lineage specification, neurite outgrowth, and maturation. This is consistent with *in vivo* localized stiffness gradients underlying regional differences and determining neural subtype, migration, and organization. As opposed to other neural modeling techniques (e.g., 3D casting of cells), refined bioprinting techniques (e.g., microfluidics) enable stiffness gradients in tissues by adjusting the concentration and flow rate of individual material components and cross-linking agents. Through 3D printing technology, controlled gradient distribution is reproducible, which is beneficial not only for consistent *in vitro* modeling of healthy tissue but allows for comparable and patient-specific heterogeneous disease modeling. Application of specific gradients is nevertheless still restricted by the current knowledge of *in vivo* topographical mapping of the brain.

In addition to a material's biological and mechanical properties to support neural tissue induction and development, its biodegradability is a key characteristic. Degradation and replacement of the material with the intercellularly synthesized ECM more closely models time-dependent developmental changes of the ECM (e.g., stiffening), which are observed *in vivo* and associated with a preferential change in neural subtype (i.e., gliogenesis). ES with electroconductive materials can augment this process, as is evident by neural activity dependent formation of the perineuronal nets surrounding neural cells and ES induced enhancement of neural ECM secretion.^169,172^ While intercellular synthesis and subsequent ECM extrusion have not been studied for previously described 3D printed neural tissue models, ECM replacement is undoubtedly connected to cellular maturation. Both maturation and functionality of these *in vitro* models require further improvement and remain in the early stages of neural tissue development. Regardless of the microenvironment, enhanced neurite outgrowth, connectivity, and maturation are consistent outcomes of *in vitro* ES of neural cells (supported by the electroconductive material) and further signify the value of ES in combination with 3D bioprinting as a neural tissue modeling platform. Nevertheless, the exact ES parameters applied are diverse and require further investigation and fine-tuning, dependent on the neural subtype and material properties (e.g., electroconductivity), further reflecting the need to implement a reproducible (e.g., bioprinted) *in vitro* scaffold, in which the interconnected factors of cell density and communication, biochemical and biophysical material characteristics must be assessed and streamlined. As such, there is potential for electrical stimulation to augment 3D bioprinted tissue constructs, toward more consistent and reproducible constructs with extensive neural networks that are more functionally mature.

## V. CONCLUSION

**FIG. 5.**
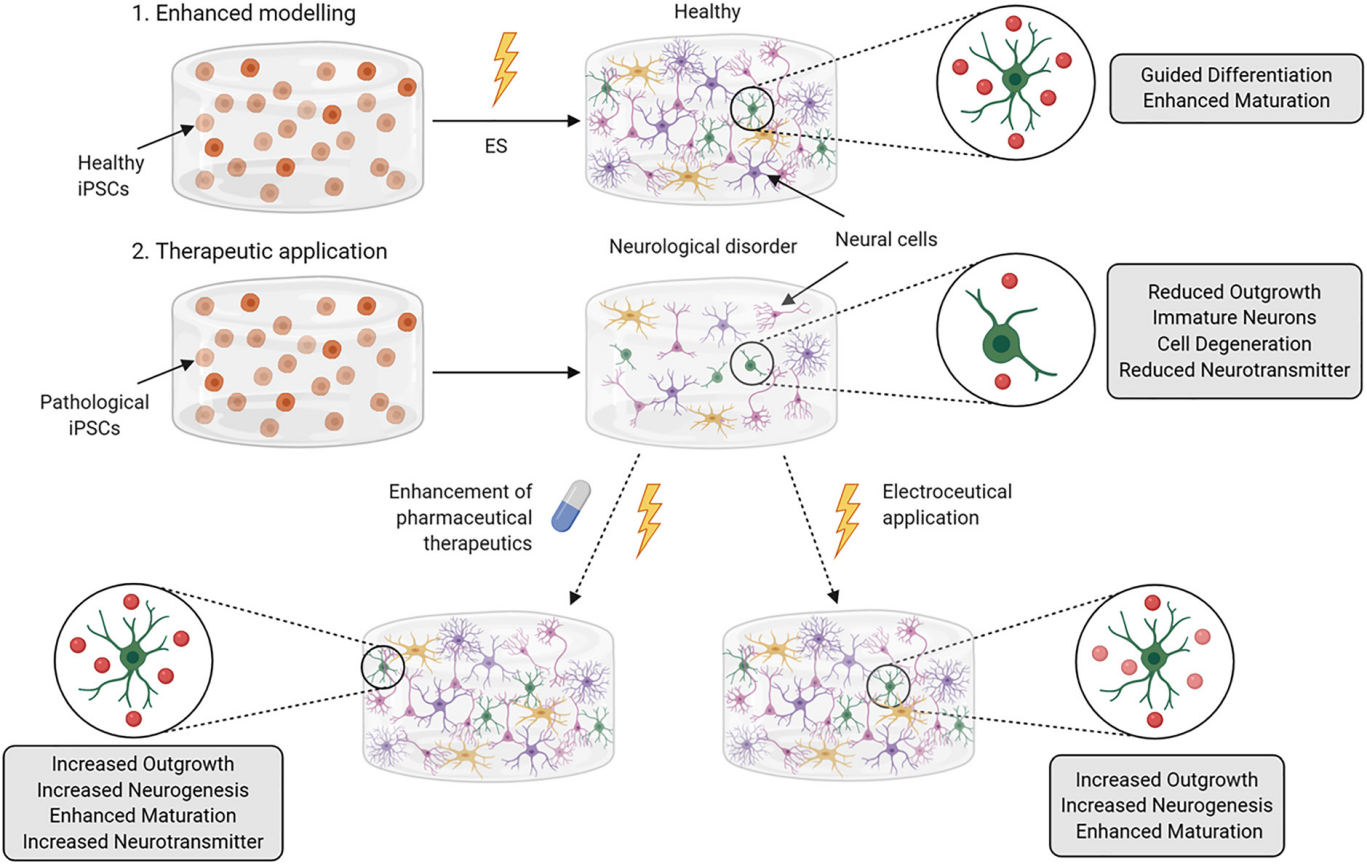
Electrostimulation as a tool for 3D tissue modeling. (1) Electrostimulation of iPSCs (and other stem cells) can be used to guide differentiation to a specific cell type and enhance neural maturation *in vitro* of engineered tissues. (2) Electrostimulation as a therapeutic tool, directly increasing neurogenesis, neurite outgrowth, and neuronal maturation, as well as augmenting pharmaceuticals potency and/or efficacy.

Given the need for clinically relevant, reproducible, and standardized *in vitro* neural tissue models for research and clinical translation, this review has sought to highlight the many facets of advanced bioprinting and the promise of ES as a means to enhance printed tissue formation, maturation, and function. In particular, biomaterials that underpin the formation of cell-supporting bioinks and act as an artificial ECM are necessary for building cell-laden scaffolds, ideally with the potential to electrically stimulate for potentially better tissue analogs. Modern practices, printer systems, and materials are conducive to ensuring improved cell viability and differentiation both during and after printing. From scaffold design to controlled temporospatial distribution of cells, *in situ* morphogenesis including neuritogenesis, and related specific cell-type functionality, there are many factors to consider for optimal and robust tissue modeling.^205^ The choice of bioprinter will depend on the desired resolution of printing, governed by the biomaterial and cells to be printed. Nevertheless, a combination of soft biomaterials and extrusion printing (including microfluidics) exhibits a low impact on iPSC/neural cell viability, and production of scaffolds with high shape fidelity has been demonstrated by cross-linking the material immediately after printing. Hydrogels are advantageous for building neural scaffolds, although somewhat constrained by batch variations of constituents materials such as alginates. Notwithstanding, they provide optimal structural stability with a low modulus, resembling the *in vivo* ECM. Moreover, combined with electroconductive properties and ES, a more rapid and potentially chemical-free approach to neural tissue induction from conductive hydrogel-laden iPSCs may even be possible (Fig. 5).

The scope for better “next generation” modeling of the intrinsic and extrinsic cellular environment is, therefore, significant, by reasonably incorporating the use of ES with 3D bioprinted materials and cells. In the case of neural tissues, this includes the optimization of relevant neural cell-subtype induction and distribution, taking into account native glial cell to neuron ratios, toward recapitulation of brain-region-specific cell communication.^167^ It is necessary to consider cellular interactions, desired or otherwise, and intrinsic cellular responses to complex physicochemical cues within the *in vivo* ECM, important to initiate, counteract, enhance, or otherwise alter neural cell and tissue development. Notwithstanding considerable knowledge of the components of the ECM, understanding of the changes during neural development is still limited. A biodegradable material that is replaced with the intercellular synthesized ECM over time should, therefore, be considered a necessity for *in vitro* neural tissue modeling. In addition, chemical cues of hormonal and immunological nature, which influence cell behavior, such as cytokines, are yet to be accounted for within *in vitro* models, despite their long-standing implication in development and diseases.^23,206,207^ With this in mind, Izsak *et al.* found that substituting adult human cerebral spinal fluid (hCSF) for common differentiation media in a 3D model of human iPSC derived neural aggregates resulted in rapid gliogenesis, neurogenesis, synapse formation, and neurite outgrowth, with the formation of mature and synchronously active neural networks, as seen in native brain tissue.^208^ Limited accessibility aside, with the aim of constructing a controlled and replicable *in vitro* tissue model, the undefined components of an individual's CSF are not desirable. However, for the formation of patient-specific and translatable cell models for *in vitro* analysis and/or therapeutic applications, it could be beneficial.

Importantly, ES for human iPSC derived neural tissue modeling also requires further study, not least of all to better define the stimulation parameters for consistent and controlled cell and tissue responses. The above-mentioned ES studies have investigated a variety of stimulation parameters to elicit a cellular response, including neural differentiation and maturation. However, it will be important to establish long-term functionality of and potentially ongoing developmental effects on complex neural networks. For example, in order to achieve native tissue-like models, it must be considered that regional differences in neural cell type distribution of the brain also apply to inherent electrical firing patterns.^209^ For this reason, region-specific responses to ES may be expected and will require careful investigation. Perhaps mimicking wide-range inherent oscillations of the sleep-wake cycle, as recorded by Llinás *et al.* and Yokio *et al.*, may provide a basis for standardized evaluation of neural responses.^173,176^

Nevertheless, current *in vitro* ES of human-derived neural tissue models illustrates enhanced modeling potential on a more translatable platform, introducing a more rapid and potentially guided induction of neural differentiation and functional maturation. As such, galvanotaxis as a response to DCEF stimulation could be a valuable platform toward neural regeneration and wound healing.^177,186,210^ Further potential therapeutic insights are gained from the example of patient-specific neurological modeling and ES application, demonstrating the ability to improve cellular pathology after 3 days of low frequency EF stimulation. This supports the use of *in vitro* ES beyond mere cellular modeling for the investigation of molecular mechanisms, toward potential patient specific adoption and clinical electroceutical application (e.g., DBS). Moreover, applied in conjunction with pharmaceutical treatment, there is further scope as a multifaceted translatable platform, through ES induced drug augmentation (Fig. 3).^211^ Disease-specific adaptation of ES to ameliorate known pathological alterations in neural network signaling and conductance holds great therapeutic promise. Further consideration and optimization of ES parameters to address disruptions to membrane potential and cellular morphology inherent to specific disease states are, therefore, imperative.^175^

Creating informative and translatable neural tissue models, representative of *in vivo* development and/or pathology, must account for potential emergent properties.^212^ Also, neurological diseases and their symptomatic presentation can vary from one individual to another, heightening the need for personalized treatment approaches. It is, therefore, crucial to be able to account for and understand these variances to control them. Notwithstanding, it is vital to develop standardized methods of building tissues through 3D printing, which account for more than the mechanical and physical characteristics and chemical environment of a scaffold, and also encompass increasingly complex materials and techniques for printing live cells, able to be artificially manipulated by, for example, ES both at the bench and *in vivo*. Such developments will indubitably overcome current challenges for engineering advanced and precise human neural tissue analogs for research and medicine.

## Data Availability

Data sharing is not applicable to this article as no new data were created or analyzed.
